# Experimental and theoretical assessments of an innovative star-shaped polyamine surfactant designed for X-65 steel corrosion mitigation in acidic environment

**DOI:** 10.1038/s41598-026-38444-4

**Published:** 2026-03-07

**Authors:** A. Elaraby, Amira E. El-Tabey, M. A. Migahed, M. Abd-El-Raouf, M. M. Shaban, E. A. Elsharaky

**Affiliations:** Egyptian Petroleum Research Institute, Nasr City, Cairo, 11727 Egypt

**Keywords:** Surfactant, Corrosion, Carbon steel, EIS, quantum calculation, Chemistry, Materials science

## Abstract

The extant study introduces newly fabricated star-shaped polyamine surfactant (*PAS*) as a highly effective corrosion inhibitor for carbon steel (*X-65*) in 1.0 M HCl environment. The *PAS* was synthesized by reaction of dodecyl amine with maleic anhydride via ring-opening reaction forming a monomaleate amide that was esterified with triethanolamine to yield a star-shaped structure, followed by Michael addition reaction using diethylenetriamine. The chemical structures of *PAS* were confirmed by FT-IR and ^1^HNMR. Surface tension measurements were employed to quantify the key surface properties of *PAS*. The inhibition performance was comprehensively evaluated through electrochemical techniques, surface characterization, and theoretical computations. Electrochemical impedance spectroscopy (*EIS*) revealed an exceptional inhibition efficiency of ~ 96% at an optimal concentration of 1000 µM, with the charge transfer resistance increasing significantly to 348.56 Ω cm^2^. Potentiodynamic polarization (*PDP*) measurements demonstrated that *PAS* operated as a mixed-type inhibitor through suppressing both anodic and cathodic reactions. The inhibition mechanism was governed by the spontaneous adsorption of *PAS* molecules onto the *X-65* surface, which follows the Langmuir adsorption isotherm suggesting a combination of physical and chemical adsorption. Surface morphology analysis utilizing scanning electron microscopy (*SEM*), energy-dispersive X-ray spectroscopy (*EDX*), and atomic force microscopy (*AFM*) provided direct evidence of the protective adsorbed film of *PAS*. Furthermore, theoretical assessments using density functional theory (*DFT*), and Monte Carlo simulations (*MCs*) successfully predicted the *PAS* active sites and its strong adsorption affinity onto the Fe surface, corroborating the experimental findings.

## Introduction

Corrosion processes lead to significant losses, particularly within industrial contexts. It is evident that prevention is the most effective strategy to mitigate these issues^1^. Various methods exist to avert or reduce the deterioration of metal surfaces. Carbon steel (*CS*), an alloy of iron and carbon, can be classified into different types based on its carbon content. The amount of carbon present plays a crucial role in determining the properties of *CS*, such as its mechanical strength, ductility, and hardness^2,3^. According to widespread availability, cost-effectiveness, besides physical and mechanical properties of *CS*, it can be applied in numerous applications across diverse industrial sectors as bridges, automobiles, machinery components, and petroleum industry^4–6^. Despite *X-65* extensive use in multiple industrial domains, it is prone to corrosion, particularly in petroleum sector during cleaning and descaling processes using corrosive acidic solution of HCl owing to its enhanced efficacy at lower temperatures and the ease of corrosion removal^7^. In economic terms, the huge damage resulted from the corrosion process in petroleum field is more than 3% of the PIB in the USA^8^. The annual direct cost of metallic corrosion ranges from 2 to 4% of the gross domestic product (GDP) in industrialized countries, and the trend is becoming higher and higher in the future^9^. A corrosion inhibitor is a widely recognized technique for controlling corrosion, utilized in minimal quantities to safeguard metals via forming a protective film layer on the surface, shielding it against the corrosive environments^10,11^. Corrosion inhibitors (*CIs*) can significantly influence *CS* corrosion process by the continuous injection into the corrosive solutions in minimal amounts, thereby hindering the interaction between the *CS* surface and corrosive agents^12^. The most prevalent and effective *CIs* are organic inhibitors, that have a variety of adsorption sites, including heteroatoms (N, O, S, and P), π-bonds, and aromatic rings within their molecular structure^13,14^**.** *CIs* with low cost have a great acceptance to be applied according to their remarkable anti-corrosive properties^15,16^. Consequently, the scientific community has initiated efforts to identify environmentally friendly inhibitors, such as organic inhibitors which utilized as cathodic, anodic, or both types of inhibitors. Generally, organic inhibitors operate through a surface adsorption mechanism accompanied with insulation film construction, and exhibit high inhibition efficiency while posing minimal environmental risks^17,18^. Organic inhibitors create a protective hydrophobic film of their adsorbed molecules on the metal surface, effectively acting as a barrier against the destructive agents in the surrounding environment^19–21^. Numerous polyamine surfactants have both amphiphilic qualities (hydrophilic and hydrophobic portions), and functional groups offering dual mechanisms of protection via reaction with metal surface forming an insulation shielding layer against the corrosive species^7,22^. Polyamine surfactants are corrosion inhibitors with distinct features, that make them useful in a variety of industrial applications. Their capacity to produce protective film, combined with minimal toxicity and environmental impact, makes them a viable alternative to standard corrosion inhibitors^9,23,24^. Shengjie Du et al.^25^ investigated the performance of HPAE-Ohs for Q235-steel inhibition in 1 M HCl solution using chemical and electrochemical techniques and the HPAE-OHs was a mixed type inhibitor with 94.9% efficiency. Wenjing Liu et al.^26^ discussed the corrosion potency of the synthesized UPy-D400-PEGDA for Q235-steel in 1 M HCl. The corrosion protection of UPy-D400-PEGDA reaches 98.80% at 500 mg/L. Punitha et al.^27^ presented the effect of PCBPE on mild steel in 1 M HCl solution in different temperatures. The protection efficiency of PCBPE enhanced with rising of concentrations till reach 90.02% at 100 ppm and diminished as temperatures increase.

The novelty of the presented manuscript is developing polyamine surfactant (*PAS*) for *X-65* corrosion mitigation with multiple amine (–NH, –NH₂) groups with lone-pair electrons that strongly adsorb onto metal surface through electrostatic attraction or coordination bonds, forming a compact barrier layer that blocks corrosive species. Besides the existence of O-atoms and ester groups (COO), decrease the metal oxidation (anodic reaction) and/or electron-consuming cathodic reactions, effectively reducing the overall corrosion rate even at very low concentrations. Also, the existence of numerous alkyl hydrophobic chains in its molecular structure help in covering and insulation of extra *X-65* surface area. This structure of *PAS* provides multiple active centers which verified the high efficiency, stable, and adsorbed protective layer of *PAS* molecules onto *X-65* surface that inhibits electrochemical corrosion reactions and prolongs the durability of metallic structures, particularly in industrial environments such as petroleum field. Unlike conventional inhibitors, *PAS* was synthesized via multi-step process specifically designed to create a 'star-shaped’ topology with a hydrophobic tail and a hydrophilic head containing numerous amine groups and displays a good solubility in polar solvents such as water, methanol, ethanol and acetone. The preparation of the studied *PAS* was achieved through the sequential combination of ring-opening, esterification, and aza-Michael addition reactions, resulting in a molecule with high adsorption capacity and multi-site binding ability on steel surfaces. The mitigation capacity of *PAS* for *X-65* type of *CS* in destructive 1.0 M HCl environment, utilizing various electrochemical techniques involved potentiodynamic polarization (*PDP*) and electrochemical impedance spectroscopy (*EIS*). Also, *X-65* surface morphology was examined prior to and following *PAS* mitigator via various surface analyses such as scanning electron microscopy (*SEM*), energy dispersive X-ray spectroscopy (*EDX*) mapping, and atomic force microscopy (*AFM*). Additionally, quantum approach was carried out employing density functional theory (*DFT*) and Monte Carlo simulations (*MCs*) predicted *PAS* active sites and its adsorption mechanisms. In this study, a wide range of concentrations of the studied *PAS* inhibitor was used for more comprehensive study starting with lower concentration (5 µM) to higher concentration (1000 µM) showing inhibition efficiency increase from 69.27 to 95.49% respectively. In practical applications, it is ideal to provide high protection with a low inhibitor dosage for economical applications.

## Experimental

### Materials

Triethanol amine, Maleic anhydride, and hexadecyl amine were obtained from Aldrich Chemical Corporation, USA. Diethylenetriamine was obtained from Fluka Chimica-Biochimica, Switzerland. All chemicals were used without further purification.

### Inhibitor preparation

**Scheme 1:**
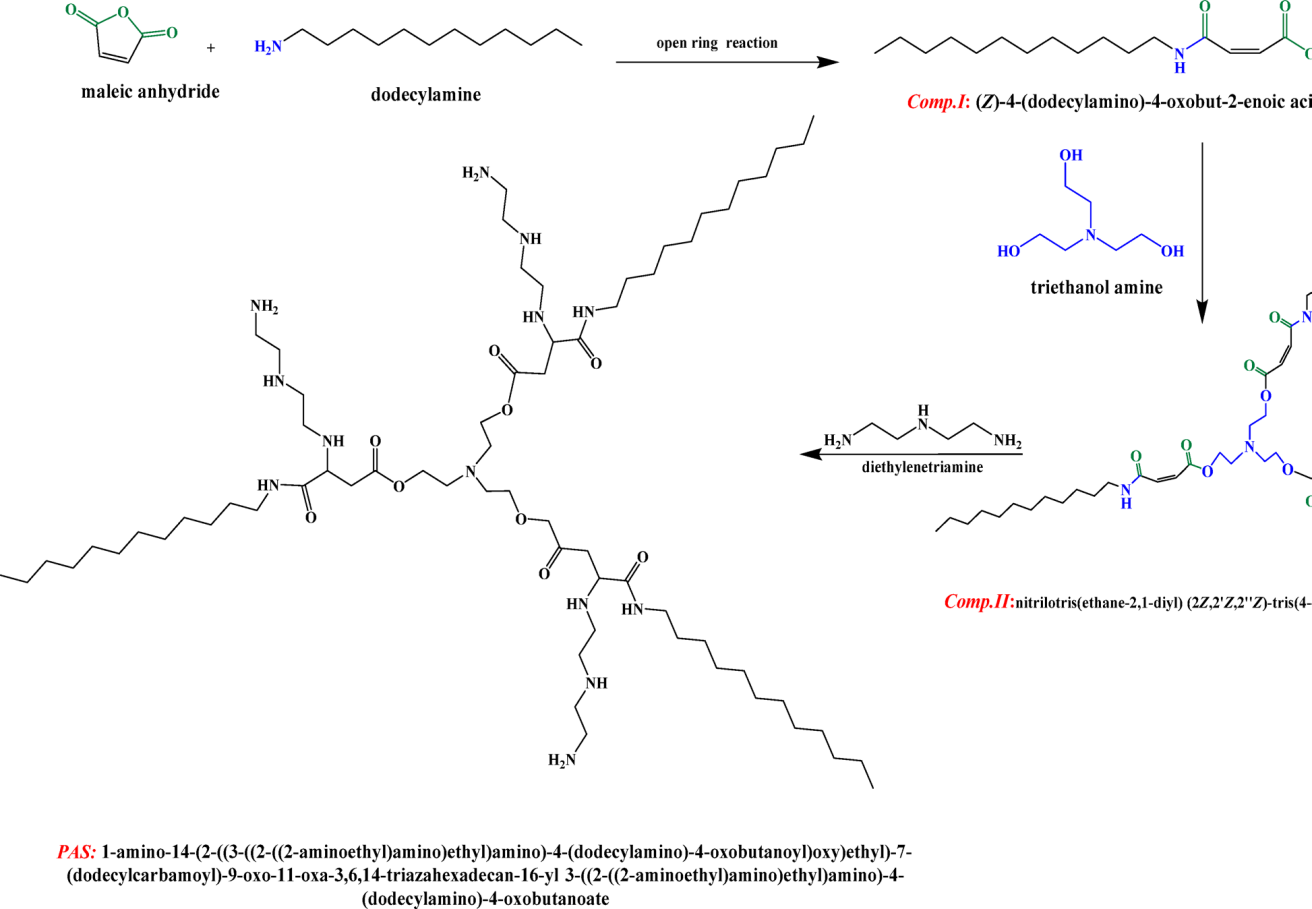
Synthesis of polyamine surfactant (*PAS*).

Three steps were applied for *PAS* preparation as observed in Scheme 1 as follow:

- *First step (Amidation reaction)*.

Maleic anhydride with 0.1 Mole was dissolved in an appropriate amount of ethyl acetate using three necked-flask with a stirrer, then 0.1 mol of dodecyl amine was added adding slowly at 50–60 °C for two hours. Subsequently, ethyl acetate solvent was distilled off to get white crystals of *Comp.I* ((Z)-4-(dodecylamino)-4-oxobut-2-enoicacid) after recrystallized from ethanol (yield 95%)^28^.

- *Second step (esterification reaction)*.

*Comp.II* (nitrilotris(ethane-2,1-diyl)(2Z,2′Z,2′′Z)-tris(4-(dodecylamino)-4-oxobu-t-2-enoate) was prepared using one neck flat bottom flask fitted with a Dean-Stark trap at 140 °C, containing 0.3 mol of prepared *Comp.I* and 0.1 mol of triethanolamine with 20 ml xylene solvent, and 2% PTSA (p-Toluenesulfonic acid) as a catalyst with continuous stirring till the theoretical water quantity was collected. *Comp.II* was obtained after purification through washing using a hot supersaturated sodium chloride solution giving organic layer was then separated, and dried utilized anhydrous sodium sulfate (yield 85%)^23^.

- *Third step (Michael addition reaction)*.

**Fig. 1:**
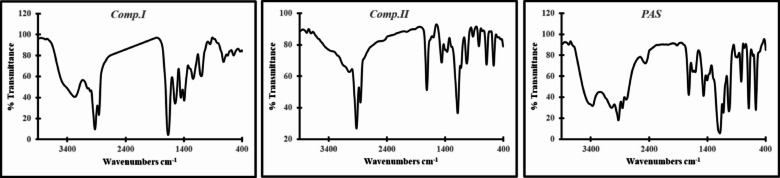
FT-IR spectroscopy of the prepared compounds.

**Fig. 2:**
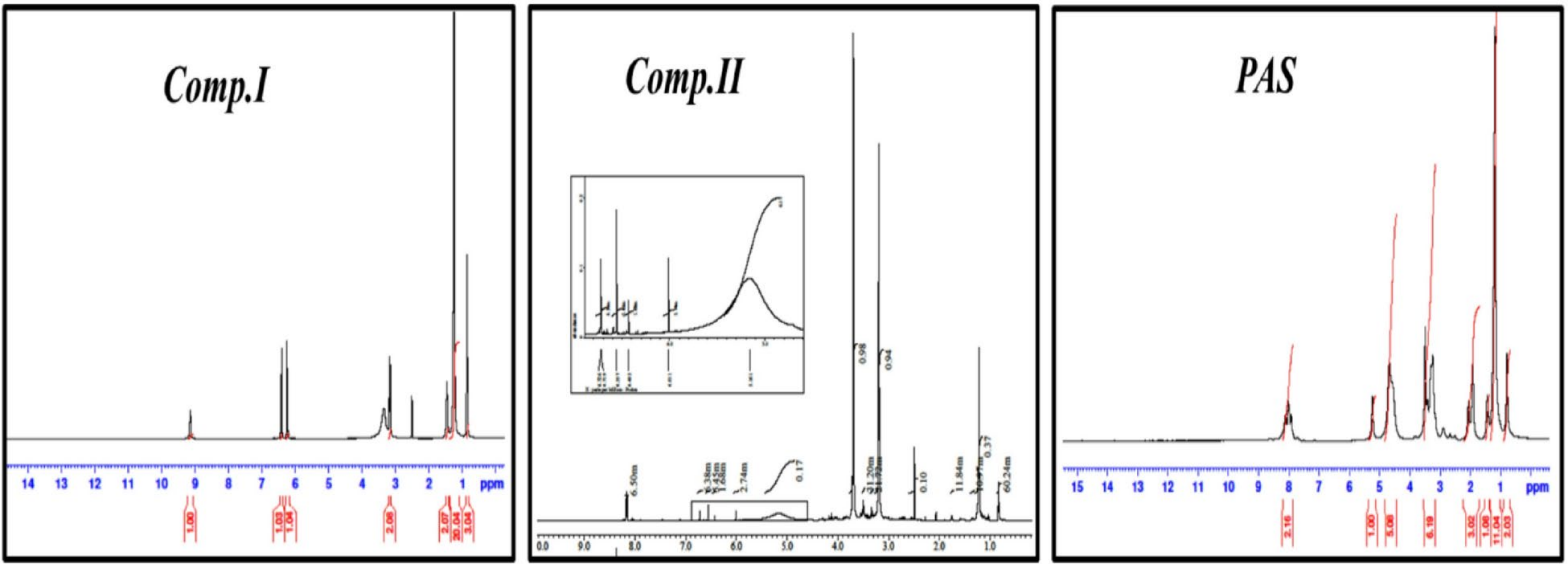
^1^HNMR spectroscopy of the prepared compounds.

Reaction mixture containing solution of (0.1 M) diethylene triamine and (0.3 M) *Comp.II* in DMF was stirred at room temperature and the reaction mixture was left overnight. Then the reaction mixture was concentrated using diethyl ether giving yellow viscous compound purified twice using chloroform^29^. The resulting compound was dried giving star-shaped *PAS* (nitrilotris(ethane-2,1-diyl)tris(3-((2-((2-aminoethyl) amino) ethyl) amino)-4-(dodecylamino)-4-oxobutanoate) (yield 85%). The chemical structure of the synthesized compounds (*Comp.I*, *Comp.II*, and *PAS*) was confirmed utilizing FT-IR and ^1^HNMR as depicted in Figs. 1 and 2 respectively.

### Surface tension measurements

Various molar concentrations of the synthesized star-shaped *PAS* were solubilized in double distilled water, and their surface tension parameters were assessed at ambient temperature (25 °C ± 1) utilizing a Theta optical tensiometer from Attension-Biolin Scientific Company.

### Electrochemical measurements

Electrochemical assays were conducted utilizing *X-65* steel as the working electrode, characterized by the following chemical composition in weight percent: 0.06% C, 0.06% Si, 0.7% Mn, 0.005% P, 0.001% S, 0.012% Ni, 0.015% Cr, 0.004% Mo, 0.002% V, and 0.02% Cu. The saturated calomel electrode (SCE) served as the reference electrode, while a The platinum wire was employed as the counter electrode, all connected to a Potentiostat-Tacussel Radiometer PGZ 402 (Volta lab 80). The experiments were performed in both the absence and presence of various doses of *PAS*. Following a 30-min open circuit potential (*OCP*) stabilization period for the *X-65* steel immersed in 1 M HCl, with and without *PAS* at different concentrations until a steady *E*_OCP_ was achieved, *EIS* was carried out over a frequency range of 10^6^ Hz to 0.05 Hz using a sinusoidal AC wave signal with a peak-to-peak amplitude of 10 mV. *PDP* curves were obtained at a scan rate of 1 mV/s, with potential sweeping ± 250 mV relative to *E*_OCP_.

### Weight loss measurements (WL)

Weight loss measurements (*WL*) of *X-65* steel were carried out at various temperature level (25 °C–55 °C) and different duration of immersion (6 h–24 h) prior to and following the injection of the optimum concentration (1000 µM) of *PAS* inhibitor.

### Surface analysis study

The detrimental impact of HCl on *X-65* steel, both prior to and following the injection of the optimum dose (1000 µM) of the synthesized *PAS*, was illustrated using *SEM* with a QUANTA FEG 250 supported with *EDX* unit, after 6-h immersion period. Additionally, *AFM* with 3D-images of *X-65* surface along the X, Y, and Z coordinate axes, before and after addition of *PAS* inhibitor were obtained using Park System XE-100.

### Computational studies

Theoretical quantum approach as *DFT* and *MCs* for the studied *PAS* inhibitor were applied utilizing BIOVIA Materials Studio 17.1.0.48 software from Accelrys, Inc according to the previous literatures^30–32^.

## Result and discussions

### Confirmation of chemical structures of the prepared inhibitors

The chemical structure of the prepared star-shaped *PAS* was confirmed by the FTIR and ^1^H NMR spectroscopy.

*The chemical structure of Comp.I was confirmed by:*

The FT-IR (KBr, cm^-1^) spectrum of the synthesized *Comp.I* presented in Fig. 1 showing a characteristic, broad absorption band at approximately 3450 cm^-1^, which is attributed to the O–H stretching vibration of the newly formed carboxylic acid group. Appearance of new bands corresponding to the amide functionality, specifically the C = O stretch (amide I band) at 1669.75 cm^-1^, stretching vibrations of the aliphatic methylene groups are observed at 2925 cm^-1^ and 2858 cm^-1^. Significantly, the spectrum shows the complete disappearance of the characteristic anhydride carbonyl bands (typically observed near 1850 and 1780 cm^-1^), which confirms the consumption of the starting maleic anhydride and the successful ring-opening amidation reaction. The ^1^HNMR (DMSO-d6) spectrum (Fig. 2), 400 MHz; δ (ppm) at 0.870 for protons of C***H***_3_; 1.242 for (C***H***_2_)n; 3.180 for C***H***_2_-NH; 6.256 and 6.433 for C***H*** = C***H***; 9.143 for amide proton N***H***.

*The chemical structure of Comp.II was confirmed by:*

The FT-IR (KBr, cm^-1^) spectrum of *Comp.II* (Fig. 1) confirms successful esterification, as indicated by the disappearance of the broad O–H stretching band at 3450 cm^-1^ and the appearance of a characteristic ester carbonyl (C=O) stretching vibration at 1714 cm^-1^. Furthermore, the presence of a C-N stretching band at 1184 cm^-1^ provides additional confirmation for the incorporation of the triethylamine moiety, confirming the complete conversion of the carboxylic acid to the ester functionality. Also, from ^I^HNMR of *Comp.II* in Fig. 2, it can be observed that, in addition to chemical shifts of monoamide maleate a new two characteristic chemical shifts were appeared at 3.19 and 3.70 of two methylene group of triethanolamine arm (NC***H***_2_-C***H***_2_O) respectively. Protons of C***H***_2_-N***H*** were shifted to lower chemical shite at 2.5 and 8.3 respectively.

*The chemical structure of star-shaped PAS was confirmed by:*

Evidence for the successful aza-Michael addition reaction is provided by the FT-IR (KBr, cm^-1^) spectrum of *PAS* (Fig. 1), which displays two key bands in the N–H stretching region: a broad band at 3369 cm^-1^ (primary amine) and another at 3100 cm^-1^ (secondary amine). Also, the disappearance of band at 1590 cm^-1^ of alkene stretching (C=C). The ^1^HNMR spectrum of star-shaped *PAS* (Fig. 2) revealed the absence of the investigative characters for the maleate vinyl protons at δ = 6.256 and 6.433, which were presented in the spectra of the precursor compounds. This disappearance is consistent with the saturation of the double bond during the conjugate addition step.

### Surface tension measurements

**Fig. 3:**
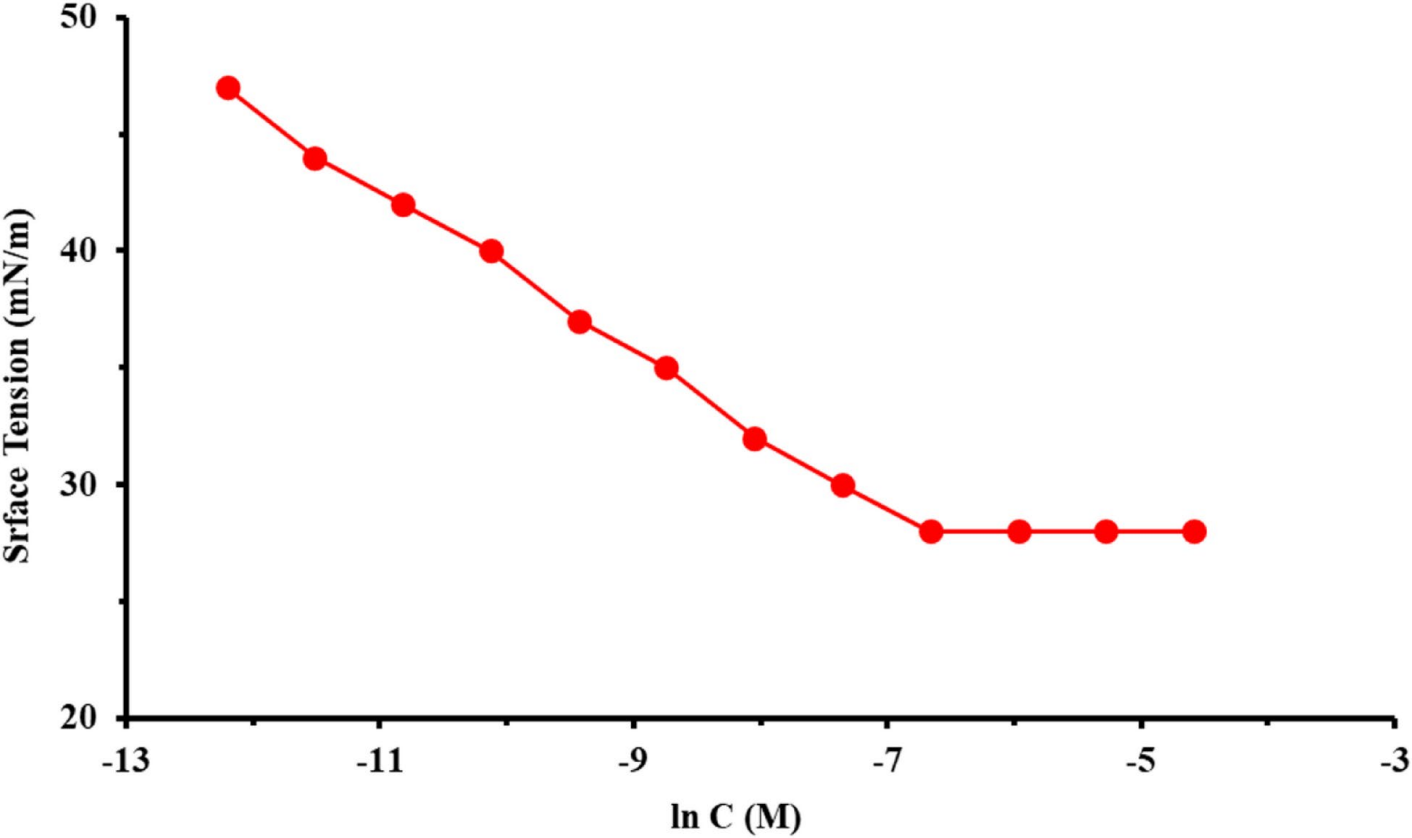
Variation of the surface tension with *PAS* concentrations at 25 °C.

The surface tension (γ) of the synthesized star-shaped *PAS* was evaluated across different concentrations, both above and below the critical micelle concentration (*CMC*) as depicted in Fig. 3, showing γ versus ln concentrations (C) of *PAS*. It was observed that the γ value exhibited a linear decrease as the concentration of *PAS* increased. This trend was consistent for the synthesized surfactant up to the *CMC*, beyond which no significant alterations were observed. The *CMC* value were determined from the inflection point in the γ–ln C graph, and documented in Table 1, which also, provides information regarding the area per molecule at the air–water interface, the effectiveness (π_CMC_), and the surface excess concentration (*Γ*_max_) of the synthesized *PAS*^33,34^. As illustrated in Fig. 3 and Table 1, the prepared surfactant effectively reduced γ of water with π_CMC_ = 28 mNm^-1^. The low surface tension value (γ_cmc_) indicated a high level of surface activity and a strong propensity for adsorption at the water/air interface^9^. The prepared *PAS* exhibits good surface properties due to its star-shaped structure, which combines hydrophobic long alkyl chains with a hydrophilic polyamine backbone. As noticed, CMC with low value (0.0025 mol L^-1^) was due to presences hydrophobic group (dodecyl group) that acted as the motivation for transitioning from the bulk solution to the interface accompanied with a decline the system’s free energy^35,36^. The adsorption of *PAS* molecules at the air–water interface can be analyzed through two key parameters: surface pressure and *Γ*_max_ that represented the peak concentration of surfactant molecules at the interface under saturation conditions. *Γ*_max_ for *PAS* was determined from the slopes of the pre-micellar region (∂γ/∂log C) as follow 37:

1 $$\varGamma _{{\max }} = \left( { - d\user2{\gamma }/d\ln C} \right)/\left( {2.303nRT} \right)$$

where, *R* and *T* denote the gas constant and absolute temperature respectively. n is the number of ions that form in solution due to surfactant dissociation. It was noticed that, *Γ*_*max*_ with lower value according to *PAS* chemical structure which enhanced its adsorption process^12,38^.

**Table 1:** Surface active parameters for the Prepared *PAS* at 25 ºC.

*Inh*	CMC (mol L^-1^)	γ_CMC_ (mNm^-1^)	П_CMC_ (mNm^-1^)	*Г*_max_ × 10^11^ (mol cm^-2^)	A_min_ (Aº^2^)	${\Delta G}_{mic}^{o}$ (kJ mol^−1^)	${\Delta G}_{\mathrm{ads}}^{^\circ }$ (kJ mol^−1^)
*PAS*	0.0025	28	44	3.22	51.45	-14.84	-14.98

Also, *Γ*_*max*_ value was utilized *A*_*min*_ (minimum area) calculation at the aqueous-air interface as follow^23^:

2 $$A_{min} = \, 10^{16} / \, N_{A} \varGamma_{max}$$

where, N_A_ = Avogadro^'^s number. The molecular area at the interface offers insights into the packing density and orientation of the adsorbed *PAS* molecules. The findings regarding *A*_*min*_ and *Γ*_*max*_ values reinforced the correlation between the adsorption capacity at solution/air interfaces and the structural characteristics of *PAS* (star-like structure), which possesses multiple active adsorption sites^9,33^. The Gibbs free energy of micellization* (*${\Delta G}_{mic}^{o}$) and ${\Delta G}_{\mathrm{ads}}^{^\circ }$ (free energy of adsorption) values were calculated and recorded in Table 1 as follow^39^:

3 $${\Delta G}_{mic}^{o}=RT\mathrm{ln}CMC$$

4 $${\Delta G}_{\mathrm{ads}}^{\circ }={\Delta G}_{mic}^{^\circ }-(6.023 \times {\pi }_{\mathrm{CMC}}{A}_{min})$$

The free energy values for ${\Delta G}_{mic}^{\circ}$ and ${\Delta G}_{\mathrm{ads}}^{\circ }$ of the synthesized *PAS* were negative, indicating that both processes are spontaneous. Furthermore, the value of ${\Delta G}_{\mathrm{ads}}^{\circ }$ was more negative than that of ${\Delta G}_{mic}^{\circ}$, suggesting that, the adsorption at the interface leads to a greater decline in the system’s free energy. This implies that the adsorption process is more favorable than micellization for the examined *PAS*^40,41^.

### Weight loss at harsh conditions

The stability of the adsorbed film of *PAS* upon *X-65* surface was investigated through weight loss (*WL*) measurements, conducted both in the absence and presence of the optimal concentration (1000 µM) across various temperatures and immersion durations.

- *Effect of immersion time*

**Fig. 4:**
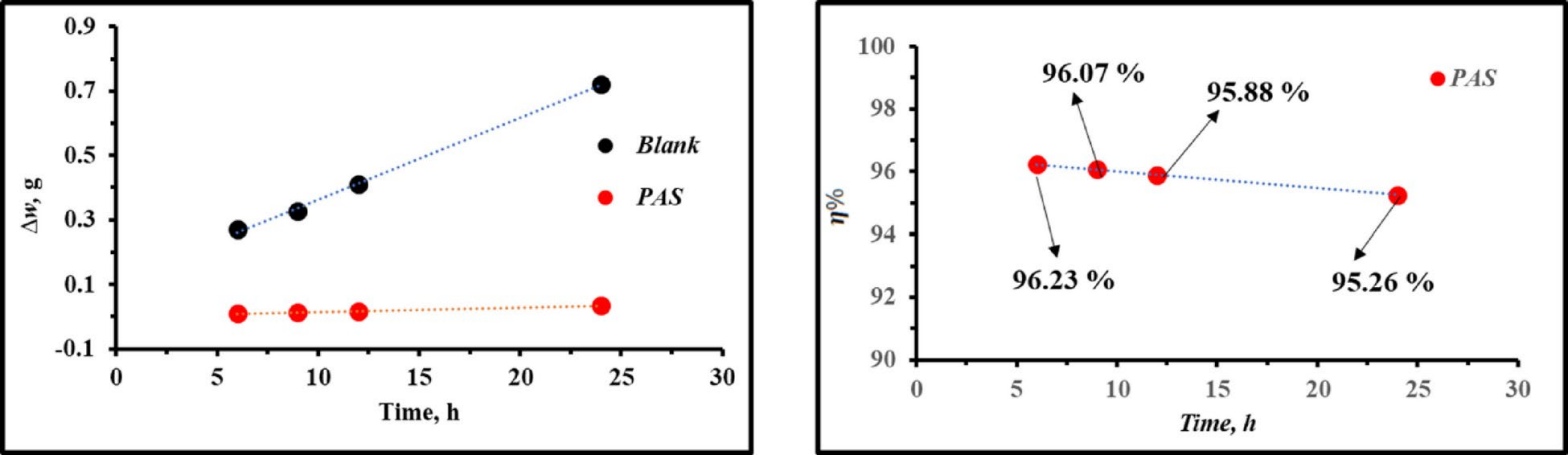
Weight difference and inhibition efficacy of the studied *PAS* vs time.

The effectiveness of the examined *PAS* inhibitor in *X-65* corrosion dominance was assessed through *WL* measurements over extended immersion periods (6 h, 9 h, 12 h, and 24 h) at 25 ºC based on the measured weights of *X-65* samples prior to and following immersion. The following equations were employed to determine the corrosion rate (*r*, g/cm^2^ h), *θ*, and *η%*:

5 $$\Delta W={W}_{o}-W$$

6 $$\theta ={W}_{o }-W/{W}_{o}$$

7 $$\eta \%=\theta \times 100$$

here, ∆*W* denotes weight difference (g). *W* and *W*_*o*_ denote the weights of *X-65* after and before immersion, respectively, measured in grams (g)^9,42^. The inhibition potency of *PAS* in Table 2 and Fig. 4 almost stable which exhibited *PAS* adsorbed film stability during prolonged immersion suggesting that, *PAS* provided a high degree of protection for *X-65* against the corrosive environments which can be elucidated by the adsorption of *PAS* molecules on the *X-65* surface forming a protective film layer^43–45^. Also, Fig. 4 presented ∆*W* over time showing blank solution with a linear relationship above that of *PAS* inhibitor with large gap among each other. As noticed in Fig. 4, ∆*W* value increased rapidly over time in the untreated corrosive solution, while after treated with *PAS*, ∆*W* value increased slightly. This observation suggested that, the formation of a protective layer of *PAS* molecules during the adsorption process on *X-65* surface, reducing the interaction between *X-65* surface and the corrosive medium, thereby mitigating the impact of the aggressive environment on the metal^46–48^.

**Table 2:** Weight loss parameters for *X-65* immersed in 1 M HCl solution with and without *PAS* at various immersion time.

*Inh*	6 h			9 h			12 h			24 h		
	∆*w,* g	*θ*	$\eta \%$	∆*w,* g	*θ*	$\eta \%$	∆*w,* g	*θ*	$\eta \%$	∆*w,* g	*θ*	$\eta \%$
*Blank*	0.2708	–	–	0.3257	–	–	0.41060	–	–	0.7216	–	–
*PAS*	0.0102	0.96233	96.23	0.0128	0.9607	96.07	0.01690	0.9588	95.88	0.0342	0.9526	95.26

- *Effect of temperature*

The influence of temperature on the stability of *PAS* film was examined across a range of temperatures (25–55 ºC) following a 6-h immersion period. The data in Table 3 indicated that, the values of *r* following the addition of *PAS* were significantly lower than those observed in 1 M HCl. This finding powered the inhibition impact of the examined *PAS* inhibitor, which can be elucidated by the attraction of *PAS* molecules to *X-65* surface through their adsorption process, leading to the formation of a protective film layer^46,49^. Furthermore, the reduction in the efficacy of *PAS* mitigator was approximately 1.86% at various temperature levels, suggesting that the effectiveness of *PAS* remains relatively stable at elevated temperatures due to the establishment of a strong protective film of *PAS* molecules upon *X-65* surface which acted as a resistant to the corrosive agents’ attacks^50–52^.

**Table 3:** Weight loss parameters for *X-65* immersed in absence and presence of *PAS* at different temperature.

*Inh*	25 ºC				35 ºC				45 ºC				`55 ºC			
	∆*w,* g	*r,* g/cm^2^	*θ*	$\eta \%$	∆*w,* g	*r,* g/cm^2^	*θ*	$\eta \%$	∆*w,* g	*r,* g/cm^2^	*θ*	$\eta \%$	∆*w,* g	*r,* g/cm^2^	*θ*	$\eta \%$
*Blank*	0.2708	0.00243	–	–	0.5283	0.00475	–	–	0.8541	0.00768	–	–	1.123	0.01010	–	–
*PAS*	0.0102	0.00009	0.9623	96.23	0.0241	0.00022	0.9543	95.43	0.0422	0.00038	0.9505	95.05	0.062	0.00056	0.9444	94.44

**Fig. 5:**
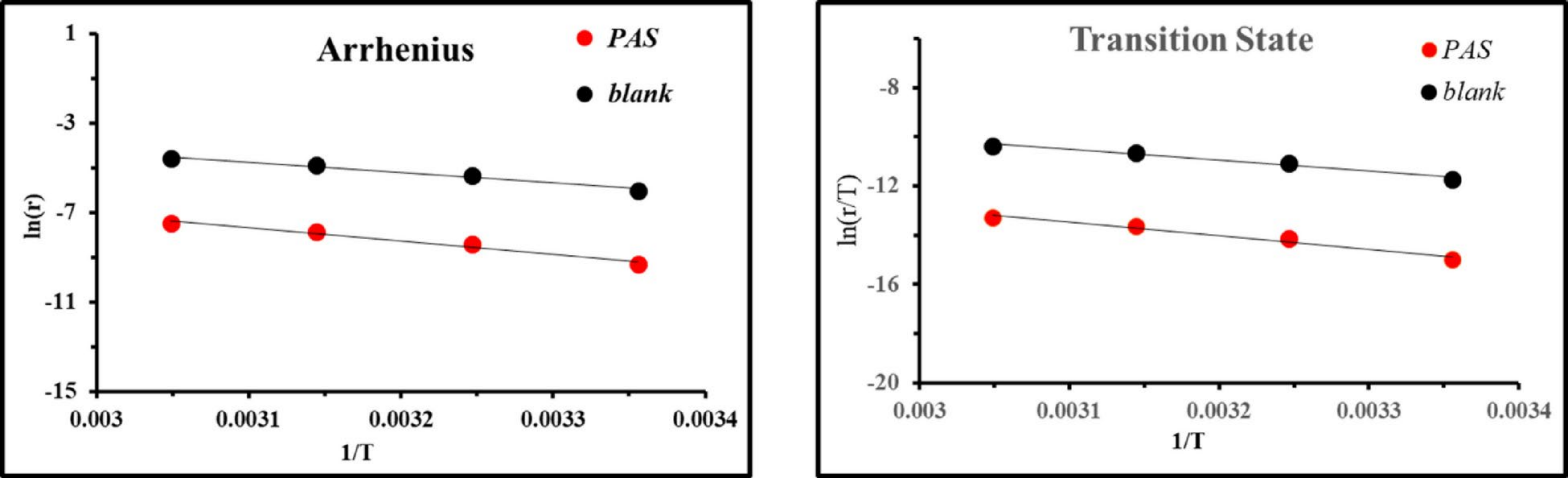
Arrhenius and transition state relations against 1/T for *X-65*in 1 M HCl free and containing *PAS* inhibitor.

Thermodynamic parameters related to activation included activation energy (*E*_*a*_), activation entropy (*ΔS*^*a*^), and activation enthalpy (*ΔH*^*a*^) were derived from Arrhenius and transition state equations (Fig. 5) based on *WL* measurements and listed in Table 4 as follow:

8 $$\text{ln }r=\mathrm{lnA}-({E}_{a}/RT)$$

9 $$\mathrm{ln}(r/\mathrm{T})=[\mathrm{ln}(R/{N}_{A}h)+(\Delta {\mathrm{S}}^{a}/R)]-(\Delta {\mathrm{H}}^{a}/\mathrm{RT})$$

here, A, R, and T represent Arrhenius constant, gas constant, absolute temperature. As well as, *h* and *N*_*A*_ denote Planck’s constant, and Avogadro’s number^8^. According to Table 4, the *E*_*a*_ value was recorded as 38.318 kJ/mol in the untreated solution, while after the introduction of *PAS*, *E*_*a*_ enhanced and reached 49.243 kJ/mol which can be attributed to an energy barrier that hinders *X-65* degradation in acidic environment, resulting in reduced mass and charge transfer^16,53,54^. Moreover, the existence of *PAS* elevated *E*_*a*_ value of *X-65*, indicating its physical adsorption through electrostatic interactions between *X-65* charged sites and *PAS* inhibitor^55,56^. *ΔH*^*a*^ values presented in Table 4 were 36.22 kJ/mol and 46.64 kJ/mol for 1 M HCL free and containing *PAS*, respectively. This observation reflected that, the corrosion process of *X-65* demands more energy than the mitigation process, and also, the positive *ΔH*^*a*^ value bolstered that, the *PAS* inhibition mechanisms was more endothermic than that of 1 M HCl solution reflecting the difficulty of *X-65* corrosion^57^. In addition, the negative *ΔS*^*a*^ value in Table 4 exhibited favorable interaction between *PAS* inhibitor and iron ions, suggesting that the stability of *PAS*–Fe complex was greater than its dissociation^58–62^.

**Table 4:** Thermodynamic activation parameters of *X-65* in absence and presence of *PAS* at different temperature.

*Inh*	Arrhenius			Transition state			
	*Slope*	R^2^	*E*_a_ (kJ mol^-1^)	*Slope*	*Intercept*	ΔH^a^ (kJ mol^-1^)	ΔS^a^ (J mol^-1^)
1 M HCl	-4669.04	0.9757	38.818	-4356.6	2.982	36.221	-172.722
*PAS*	-5922.97	0.9671	49.243	-5610.5	3.927	46.645	-164.869

### Electrochemical impedance spectroscopy (EIS)

**Fig. 6:**
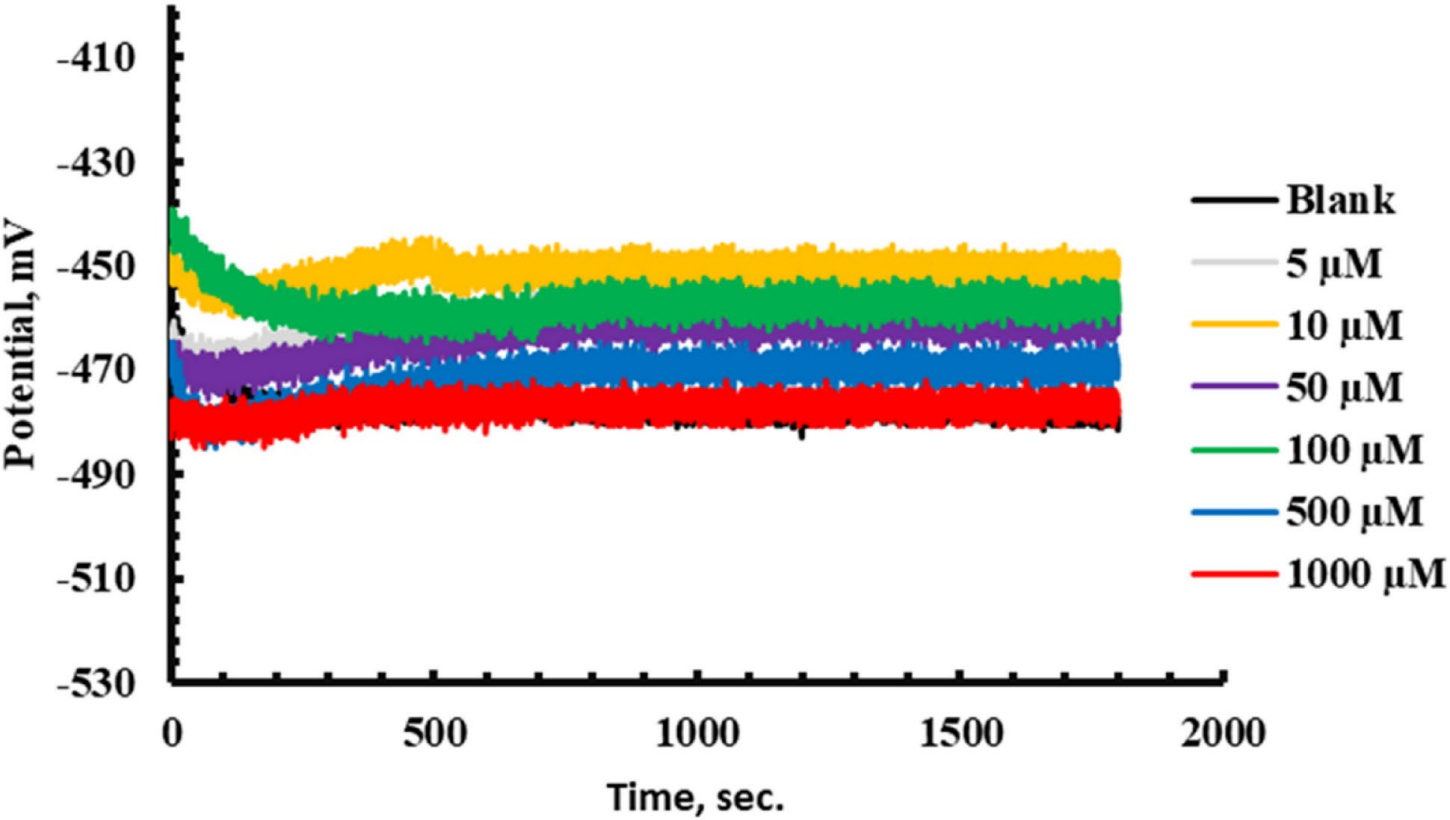
OCP Vs Time for *CS* in 1 M HCl in absence and presence of different concentrations of *PAS* inhibitor at room temperature.

**Fig. 7:**
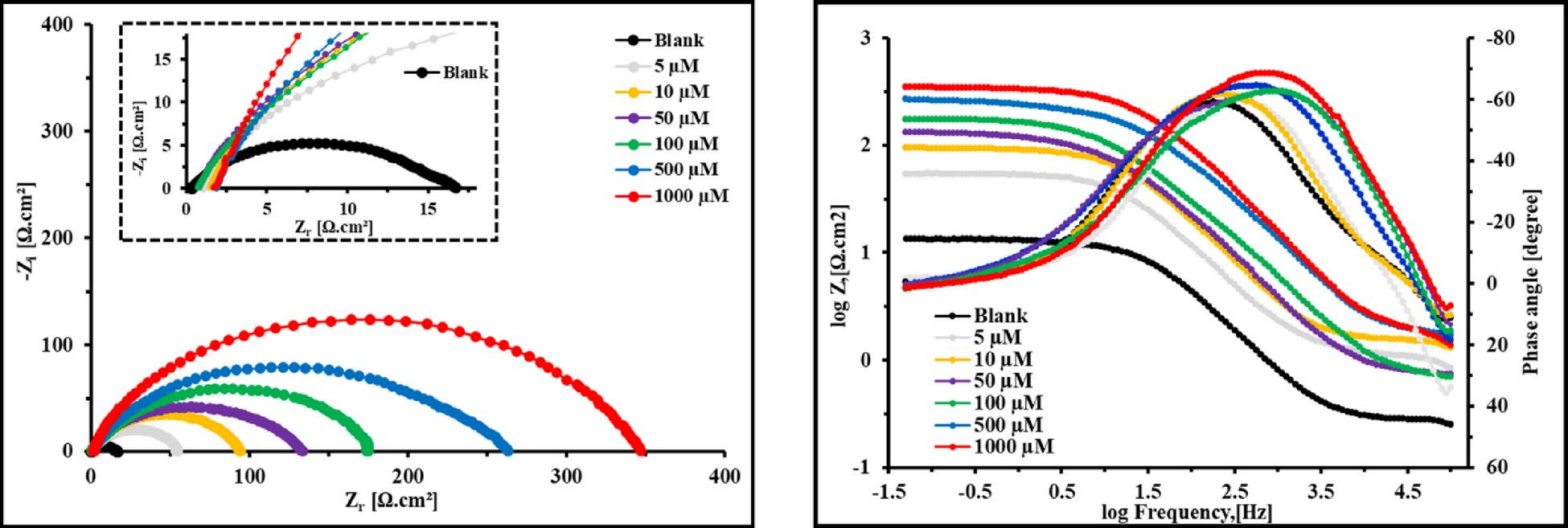
Nyquist and Bode-phase curves of *X-65* in 1 M HCl without and with different concentrations of *PAS* inhibitor.

**Fig. 8:**
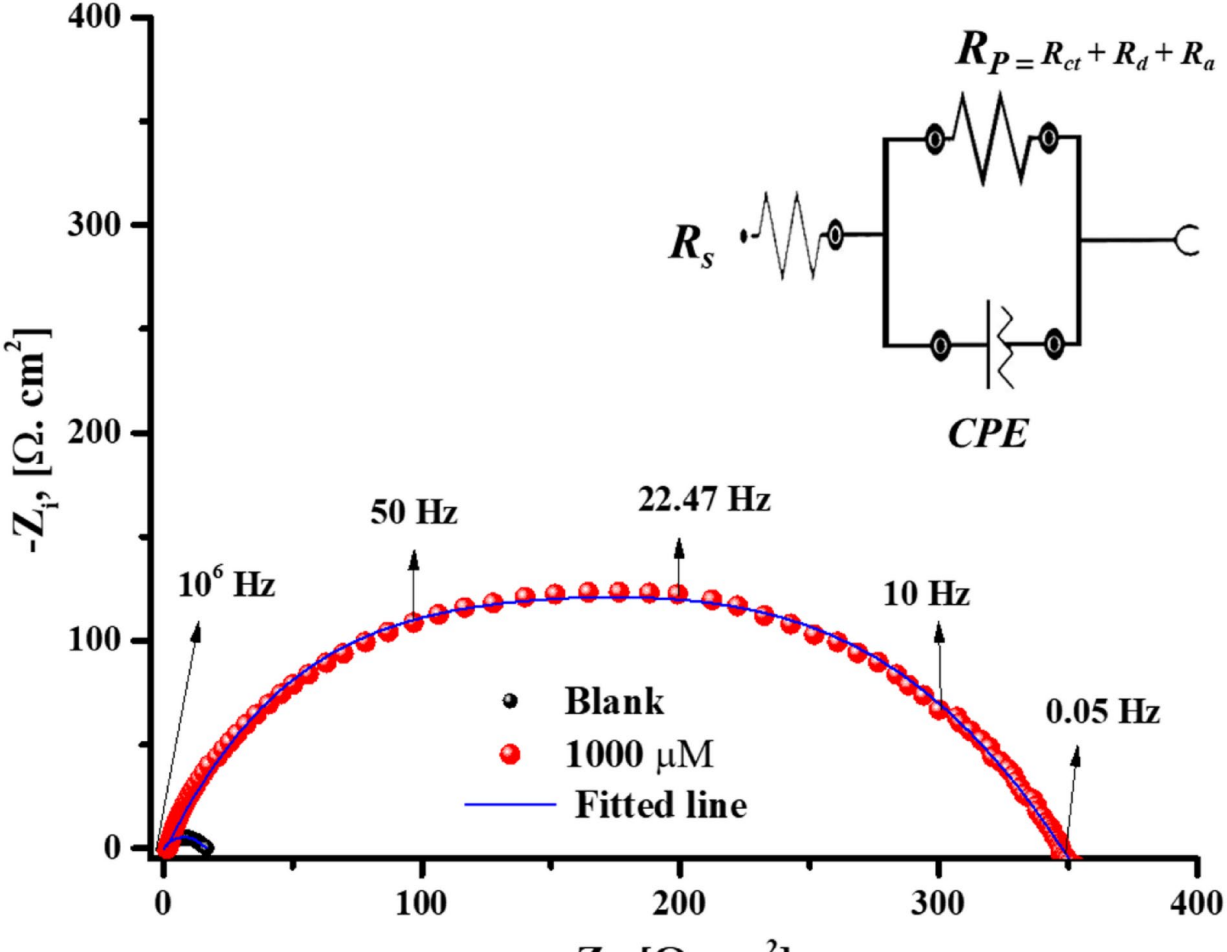
Nyquist plots of *X-65* in 1 M HCl in absence and presence of 1000 μM of *PAS* inhibitor using the proposed equivalent circuit.

It is imperative to achieve a steady state process before starting Electrochemical assays. *E*_ocp_ variation of *X-65* with time in 1 M HCl free and containing different doses of *PAS* inhibitor until the steady-state potential was depicted as seen in Fig. 6. It was observed that, OCP tends to be stable with time and less fluctuates were observed. Also, the rapidly and negligible variations in the OCP can be attributed to the modification of the *X-65* surface with the adsorbed *PAS* molecules with a steady-state had been achieved after 300 s. This findingexhibited that, *PAS* has more thermodynamically stable state and effective adsorption over *X-65* surface. Also, the introduction of *PAS* inhibitor shifted *E*_ocp_ value with very low alteration, confirming that the investigated *PAS* operated as mixed-type inhibitor via blocking both *X-65* cathodic and anodic sites^63,64^. *EIS* is a widely used technique in corrosion studies using AC (Alternative Current) and wide range of frequencies for kinetic and mechanistic information at equilibrium (steady state) of the electrochemical system^65^. At room temperature (25 °C), the electrochemical behavior of *X-65* was assessed in 1 M HCl environment with and without various quantities of the prepared *PAS* as shown in Fig. 7, showing Nyquist spectra and its corresponding impedance parameters as in Table 5. As noticed in Fig. 7, a significant increase in the diameter of *X-65* Nyquist arcs after the addition of *PAS* relative to that of HCl free bolstered the anticorrosion effect of *PAS* and its surface coverage ability via construction of a protective film against HCl solution^66–68^. Also, the enhancement of Nyquist plots diameter with *PAS* concentrations is related to film thickness rising owing to adsorption of more quantities of *PAS* molecules^69^*.* This observation confirmed the mitigation impact of the studied *PAS* and *X-65* degradation mechanism was regulated by charge transfer process which was also assured from bode curves as shown in Fig. 7 proffering enhancement in the gab distance between bode-phase curves of the untreated solution with *PAS* concentrations^70–72^. The shift in bode curves to higher value at various doses of *PAS* relative to blank solution (1 M HCl) demonstrated the adsorption power of the prepared *PAS* which was also confirmed by the appearance of phase curves of *PAS* towards – 90° compared with blank solution as seen in Fig. 7^73,74^. All these annotations reflected the mitigation power of the prepared *PAS* through its adsorption process and shielding *X-65* surface against the ruinous species of the surroundings environment^75^. Nyquist arc looked as imperfect capacitive loop unlike an ideal capacitor which can be attributed to frequency dispersion phenomena and *X-65* surface heterogeneity as well diameter alteration between *PAS* molecules and electrons at *X-65*/solution interface, as both of + ve and -ve charges of *PAS* molecules and electrons respectively are equal^76^. It was observed that, a simple Randles EC (Equivalent Circuit) with one time constant was applied for *X-65*/electrolyte interface definition as in Fig. 8 involves resistance of electrolyte (*R*_S_), constant phase element (*CPE*), and polarization resistance (*R*_P_) which involved *R*_ct_ (charge transfer resistance), *R*_d_ (diffuse layer resistance) and *R*_a_ (accumulation resistance) in absence of *PAS* inhibitor. While, in the existence of *PAS*,* R*_P_ involved *R*_*ct*_, *R*_*d*_, *R*_*a*_, and *R*_*f*_ (film resistance)^31^. Additionally, *X-65* heterogeneity was studied using *CPE* instead of *C*_dl_ (Double Layer capacitance) that was clarified using two factors, *Y*^°^ (*CPE* magnitude) and *n* (phase shift) according to the following equation^37^:

10 $${Z}_{CPE}={Y}_{0}^{-1}(j{\upomega }_{max}{)}^{-n}$$

here, *j* and *ω* are imaginary root and angular frequency respectively. While *C*_dl_,* τ* (relaxation time), and *T* (Thickness) can be computed using the following equation:

11 $${C}_{\mathrm{dl}}=\left(\frac{{\varepsilon }^{^\circ }\varepsilon }{T}\right)A$$

12 $${C}_{\mathrm{dl}}=1/(2\uppi {R}_{ct}{F}_{img\to Max})$$

13 $$\uptau ={C}_{\mathrm{dl}}\times {R}_{\mathrm{ct}}$$

here, ${F}_{img\to Max}$, *A,* ε^◦^, and ε denote the frequency at maximum imaginary impedance, *X-65* surface area, air permittivity and dielectric constant respectively^67^. According to the above equation, the presence of *PAS* decreases the value of *C*_dl_ which was also emphasized from *Y*^°^ value that drops with *PAS* concentrations rising^77,78^. This fact designated the adsorption power of *PAS* due to its special structure over *X-65* surface via H_2_O replacement process by *PAS* molecules consequently, enhanced the film thickness and *X-65* surface coverage by reducing the exposed *X-65* surface area to the destructive environment^79^. The rising values of coefficient n following the introduction of PAS inhibitor, as illustrated in Table 5, indicated an improvement in *X-65* surface homogeneity owing to the adsorption and accumulation arrangement of *PAS* molecules at the interfaces of the *X-65*/HCl solution, as well as the energy distribution within the film layer^80^. Also, the introduction of the examined *PAS* inhibitor enhanced *τ* to higher value as observed in Table 5, indicating that, *PAS* molecules are gradually adsorbed onto *X-65* surface, leading to construction of a stable film layer of *PAS* molecules that effectively shielding *X-65* surface from the destructive species^81^.

**Table 5:** EIS parameters of for X-65 immersed in 1.0 M HCl without and with various doses of PAS inhibitor.

*Inh*	*Conc.* (µM)	*R*s, (Ω cm^2^)	*CPE*		*C*_dl_ (F/cm^2^) × 10^–5^	*τ * (s)	*R*_P_ (Ω cm^2^)	*θ*	$\eta \%$
			*n*	*Y°* (µ*S*/cm^2^)					
Blank	–	1.391	0.991	107.23	2.811	0.00445	15.86		–
*PAS*	5	1.724	0.981	62.91	2.196	0.01133	51.62	0.6927	69.27
	10	2.341	0.994	54.86	1.383	0.01273	92.06	0.8277	82.77
	50	2.138	0.989	48.02	1.239	0.01592	128.45	0.8765	87.65
	100	1.097	0.976	39.45	1.034	0.01783	172.37	0.9079	90.79
	500	1.781	0.969	23.18	0.301	0.00796	265.04	0.9397	93.97
	1000	1.139	0.998	13.09	0.204	0.00713	348.56	0.9546	95.46

The following equations were used to estimate the *θ* and *η%* of the prepared *PAS* based on* R*_P_ value:

14 $$\theta =({R}_{P. PAS}-{R}_{P. blank})/{R}_{P. PAS})$$

15 $$\eta \%=\theta \times 100$$

where, ${R}_{P. blank}$ and ${R}_{P}$ are the polarization resistance in absence and presence of *PAS* respectively^82^. From Table 5, the high values of *R*_P_ and *η%* in the existence of *PAS* reveals its mitigation power and significant role in *X-65* protection. The *R*_*b*_ value increased to 348.56 Ω cm^2^, compared to a baseline of 15.86 Ω cm^2^ for the blank, at an optimum concentration of 1000 µM PAS. This is a direct result of the inhibitor’s branched, star-shaped molecular design. This unique geometry enables multi-point adsorption, where numerous functional groups (heteroatoms N, O and C = O groups of esters) from a single molecule can bind to the surface synchronously. This leads to the formation of a strong and extensive defensive layer that significantly inhibits corrosion. Also, *η%* enhanced with *PAS* doses till touch 95.46% at 1000 µM. All these annotations bolstered the perfect surface coverage of *PAS* molecules over *X-65* surface and significant reduction of *X-65* corrosion after the addition of *PAS* consequently drop *X-65* degradation rate^83,84^. The experimental corrosion data obtained from *EIS* and *PDP* exhibited a strong correlation with each other.

### Polarization measurements (PDP)

**Fig. 9:**
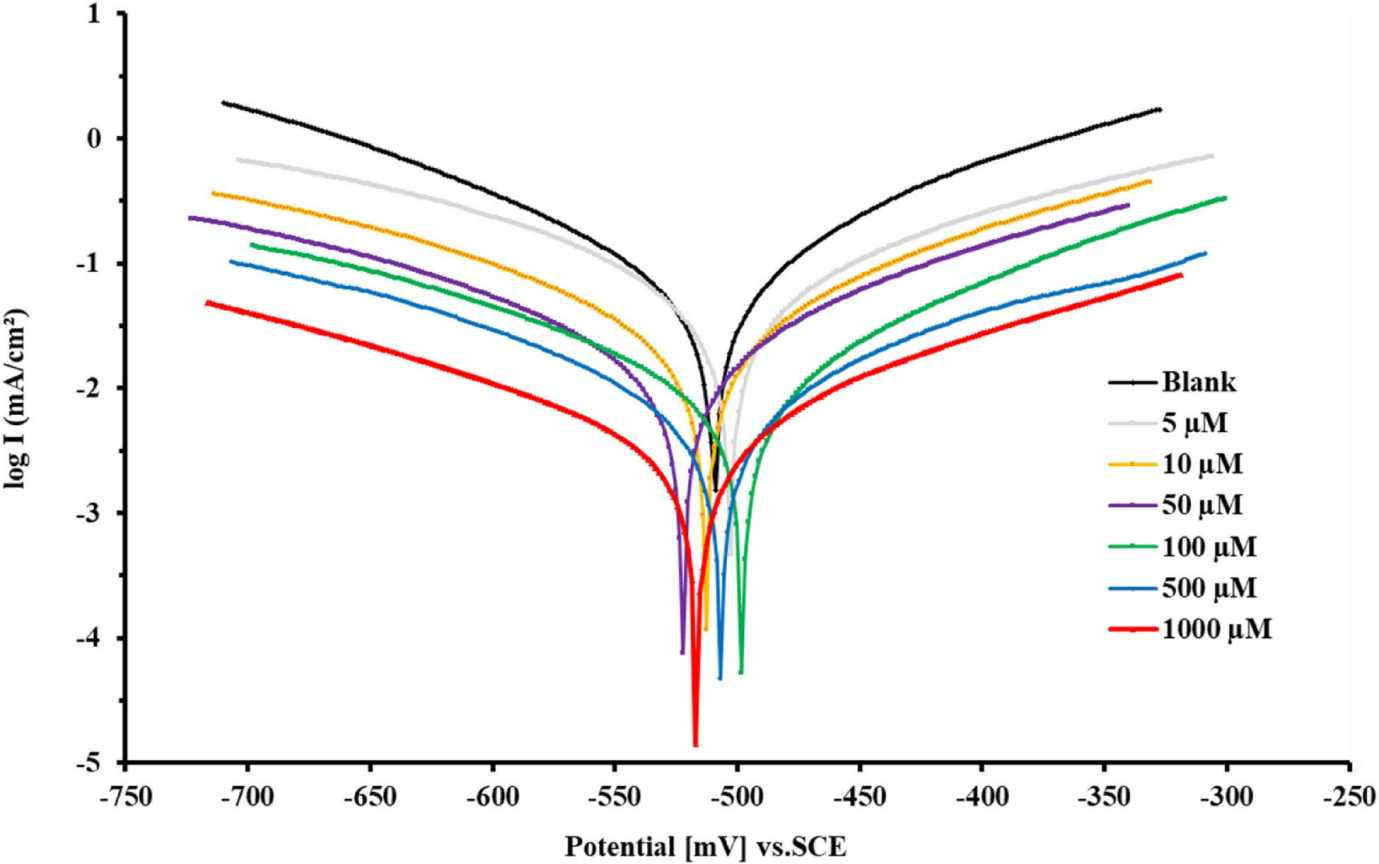
*PDP* curves for *X-65* in 1 M HCl with and without different concentrations *PAS* at 25 °C.

*PDP* analysis provides valuable information about the electrochemical kinetics associated with corrosion and mitigation mechanism. *PDP* diagrams of *X-65* immersed in 1 M HCl Prior to and following *PAS* addition were illustrated as seen in Fig. 9. The addition of *PAS* mitigator retarded both the anodic *X-65* corrosion and cathodic H_2_ production owing to the blocking ability of the studied *PAS* inhibitor. Also, a notable shift in Tafel curves to more negative values in lower current density region which influenced by concentration rising of *PAS* according to its adsorption process upon *X-65* surface accompanied with a decline in Cl^-^ and H^+^ in the corrosive medium^65^. The same appearance of Tafel curves parallel lines reflected that, *X-65* corrosion mechanism didn’t alter with the existence of *PAS* inhibitor. Besides, the reduction of hydrogen reaction was governed by the adsorption of *PAS* molecules over *X-65* forming insulation barrier layer between *X-65* surface and the destructive surrounding subsequently, reduced the available surface area for the adsorption and reduction of hydrogen ions^85^. The protection process of *X-65* surface can be elucidated by the adsorption of the prepared *PAS* and formation of an insulation defensive layer from the adsorbed *PAS* molecules against the destructive particles subsequently suppressed *X-65* corrosion rate^86^. Some associated corrosion parameters include *i*_corr_ (corrosion current density) and corrosion potential (*E*_corr_), along with both the Tafel anodic (*β*_a_) and cathodic (*β*_c_) slopes were tabulated as in Table 6. Utilizing the obtained *i*_corr_ value, *θ* (Surface Coverage) and inhibition efficacy (*η%*) values were calculated and are also displayed in Table 6 as follow^87,88^:

16 $$\theta =({i}_{\mathrm{corr}. HCl}-{i}_{\mathrm{corr}. PAS})/{i}_{\mathrm{corr}.\mathrm{HCl}})$$

17 $$\eta \%=\theta \times 100$$

here, ${i}_{\mathrm{corr}. HCl}$ and ${i}_{\mathrm{corr}. PAS}$ are the uninhibited and inhibited corrosion current densities respectively. The obtained data in Table 6 reflected that, the prepared *PAS* operated as mixed-type inhibitor which can be confirmed from *E*_corr_ value with very low alteration prior to and following *PAS* introduction via blocking both *X-65* cathodic and anodic sites which can be also proved from* β*_a_ and *β*_c_ value^12,89^. Also, the existence of *PAS* decreased *i*_corr_ till reach 0.0396 mAcm^−2^ at 1000 µM compared with that value of the unprotected solution 0.7381 mAcm^−2^ with inhibition efficiency touched 94.63%. This annotation is explained by the strong adsorption of the prepared PAS at the X-65 surface, due to the molecule’s star-shaped design. This structure inherently provides multiple active centers for adsorption, including ester groups, amide groups, and the extensive polyamine chain. The star shape allows these groups to adsorb at the surface simultaneously, leading to enhanced adsorption kinetics and high inhibition efficiency^37,74^.

**Table 6:** PDP parameters for X-65 immersed in 1.0 M HCl without and with various doses of PAS inhibitor.

Inh	Conc., (µM)	*** − E***_corr_ (mV)	***i***_**corr**_ (mAcm^**−2**^**)**	***β***_**a**_ (mVdec^-1^)	** − *β***_**c**_ (mVdec^-1^)	***θ***	${\boldsymbol{\eta}}{\%}$
Blank	–	509.2	0.7381	107.2	149.5	–	–
*PAS*	5	502.3	0.2813	98.7	161.9	0.6188	61.88
	10	513.1	0.2274	108.1	143.7	0.6919	69.19
	50	521.9	0.1479	114.6	179.5	0.7996	79.96
	100	498.7	0.1197	138.5	163.4	0.8378	83.78
	500	506.8	0.0723	96.9	171.4	0.9021	90.21
	1000	517.3	0.0396	108.6	181.2	0.9463	94.63

### Adsorption isotherm

**Fig. 10:**
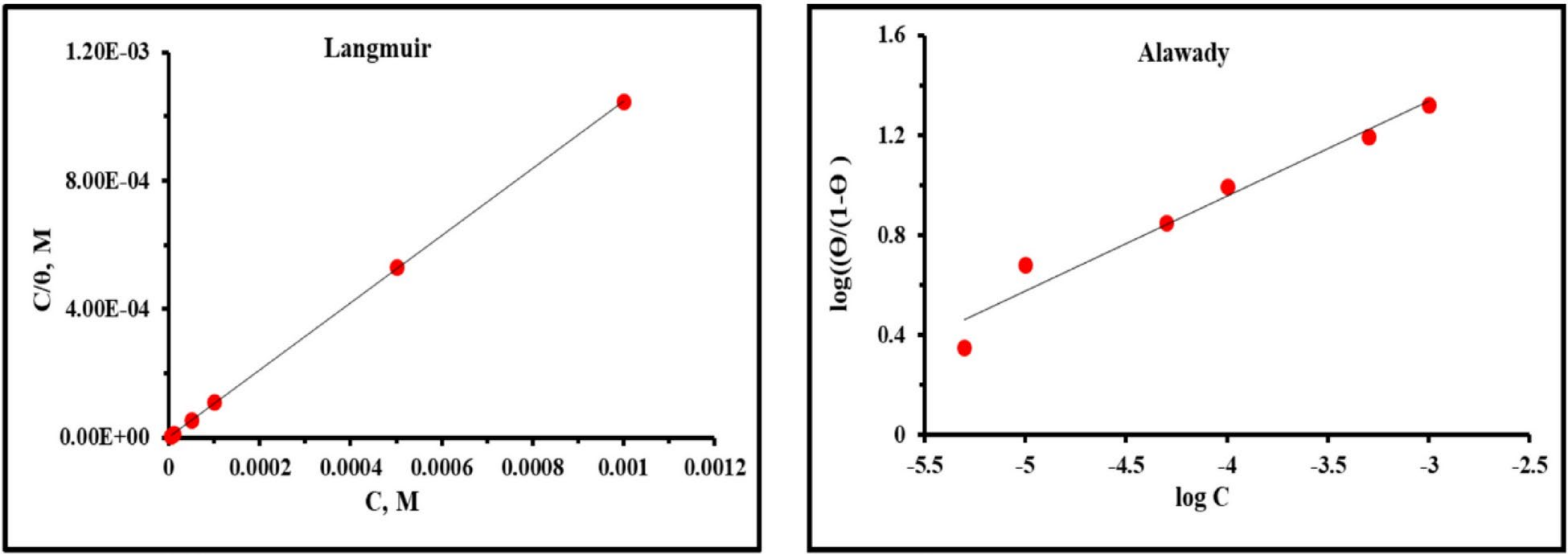
Langmuir and Alawady isotherms of *PAS* adsorption at *X-65*/HCl interface using EIS at room temperature.

The adsorption of *PAS* molecules upon *X-65* surface can be considered as a substitution process with water the adsorbed water molecules^90,91^. Many isotherms were applied using *EIS* data to find the most fitting isotherm for *PAS* adsorption reflecting Langmuir isotherm was the suitable one with regression coefficient (R^2^ = 0.9999) and linear relation of C/θ vs. C as depicted in Fig. 10, giving a slope close to unit and intercept equal to (1/*K*_ads_) according to the following equation:

18 $$^{\circ}\mathrm{C}/\uptheta=(1/{K}_{\mathrm{ads}})+\mathrm{C}$$

here, *K*_ads_ denotes the adsorption equilibrium constant^92^. Langmuir isotherm posits that there is no interaction among the adsorbed molecules, suggesting that the adsorption energy remains constant regardless of *θ*. It also postulated that metal surface has a fixed number of adsorption sites, with each site accompanied with a single adsorbed species^93–95^. The large *K*_ads_ value in Table 7 revealed the ease and strong adsorption of *PAS* molecules upon *X-65* surface shielding it against the destructive agents and formation of a protective barrier layer though *PAS* adsorption via numerous hetero atoms in its molecular structure^96^. The analysis of the Langmuir isotherm slope value, which exceeds 1, indicates that the adsorption of *PAS* on *X-65* surface is more effectively described by a modified Langmuir equation known as Villamil isotherm. This model implies that each unit of *PAS* occupies multiple adsorption sites, besides interactions among the adsorbed *PAS* species over *X-65* surface that accompanied with extra surface coverage as follow^97,98^:

**Table 7:** Langmuir and Alawady parameters of *PAS* adsorption at *X-65*/HCl interface using *EIS* data at room temperature.

*Inh*	Langmuir				Alawady			
	R^2^	Slope	*K*_ads_ (L mol^−1^)	${\Delta G}_{\mathrm{ads}}^{\circ }$ (kJ mol^−1^)	R^2^	*y*	*K*_ads_ (L mol^−1^)	${\Delta G}_{\mathrm{ads}}^{\circ }$ (kJ mol^−1^)
*PAS*	0.9999	1.046136	246,740.7	-40.712	0.9591	0.379935	3,289,188.85	-46.338

19 $$C/\theta=nC+(n/{K}_{ads})$$

In this context, n represents the calculated slope value which denotes the quantity of water molecules adsorbed during displacement^99^. Furthermore, the experimental data were analyzed using the Kinetic adsorption isotherm model proposed by Alawady, as described by the following equation^100^:

20 $$\mathrm{ln}(\theta/1-\theta )=\text{ln }K+y\text{ln }C$$

here, *K*′ and *y* are constants associated with *K*_ads_ (where, *K*_ads_ = K^1/y^), and the number of *PAS* molecules that occupy single active site. The linearity of Alawady isotherm in Fig. 10 gives a slope equal y and intercept equal ln(K′). The value of *y* (< 1) as in Table 7 reflected that; *PAS* molecules occupied multiple active sites^101^. Furthermore, since *y* value < 1, suggesting the formation of a monolayer on the metallic surface, consistent with the principles of the Langmuir adsorption isotherm. Also, *R*_*L*_ (dimensionless separation factor) was calculated based on *K*_ads_ value obtained from Langmuir and Alawady isotherms as the next equation:

21 $${R}_{l}=1/(1+{K}_{ads} C)$$

As observed, *R*_*L*_ in Table 8 with a small value less than unit (*R*_*L*_ < 1) reflecting the high adsorption capacity of the studied *PAS* on *X-65* surface^23^. $\Delta {G}_{\mathrm{ads}}^{^\circ }$ in Table 7 can be calculated established on *K*_ads_ value as follow^38,79^:

22 $$\Delta {G}_{\mathrm{ads}}^{^\circ }=-\mathrm{RTln}(55.5{ K}_{\mathrm{ads}})$$

here, 55.5 denotes water concentration (mole L^−1^). In general, $\Delta {G}_{\mathrm{ads}}^{^\circ }$ with value higher than − 20 kJ mol^−1^ corresponded to the electrostatic reaction between charged molecules and charged metal (physical adsorption), while with value lower than − 40 kJ mol^−1^ involved the electron sharing from the inhibitor molecules to the metal surface forming coordination bond (chemisorption)^102,103^. It was found that, the value of $\Delta {G}_{\mathrm{ads}}^{^\circ }$ was lower than − 40 kJ mol^−1^, demonstrating that, the adsorption mechanism of prepared *PAS* upon *X-65* surface was chemisorption process. In addition, the -ve sign of $\Delta {G}_{\mathrm{ads}}^{^\circ }$ value tabulated in Table 7 indicated that, *PAS* molecules adsorbed over *X-65* surface spontaneously^104,105^.

**Table 8:** Values of *R*_L_ for *X-65* immersed in presence of various concentrations of *PAS* in 1 M HCl solution.

*Conc.* (µM)	Langmuir		EIS	
	*PDP*	*EIS*	*PDP*	*EIS*
	*R*_L_	*R*_L_	*R*_L_	*R*_L_
5	0.6567	0.4476	0.2493	0.0573
10	0.4888	0.2884	0.1424	0.0295
50	0.1605	0.0749	0.0321	0.0060
100	0.0873	0.0389	0.0163	0.0030
500	0.0187	0.0080	0.0033	0.0006
1000	0.0094	0.0040	0.0016	0.0003

### Quantum chemical calculations

#### DFT (density functional theory)

**Fig. 11:**
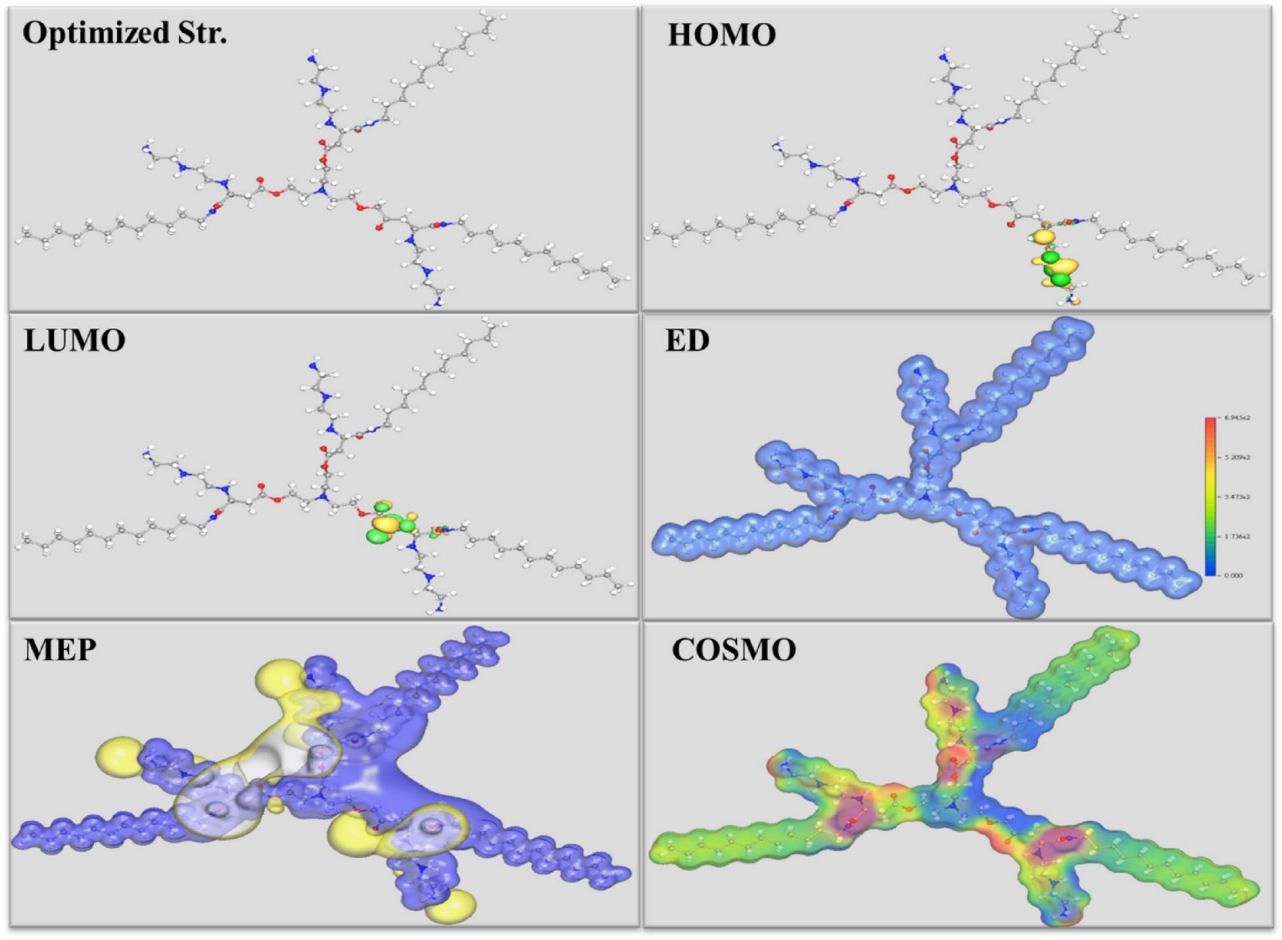
Optimized structures, HOMO, LUMO, ED, MEP, and COSMO field of the studied *PAS*.

The optimized molecular configuration of the prepared *PAS* in aqueous environment was presented as depicted in Fig. 11 showing its FMOs (Frontier Molecular orbitals) containing HOMO (Highest Occupied Molecular Orbital) and LUMO (Lowest Unoccupied Molecular Orbital) regions which are responsible for *PAS* reactivity and electronic characteristics. The appearance of *PAS* with a planar situation with the *X-65* surface, providing more *X-65* surface protection^106–108^. The HOMO distributions represented the nucleophilic centers in Fig. 11 were localized on amino side chain with several -NH groups and ethylene spacers which highlighted coordination bond formation via electron sharing process to the unoccupied 3d-orbitals of iro^109,110^. While, the LUMO distributions represent the electrophilic centers were localized on carbonyl group, amide group and O-atoms which exhibited the ability of *PAS* to acquire electrons from *X-65* surface via back donation process^49,111,112^. Also, the reactivity of the prepared *PAS* was confirmed through ED (Electron Density) and MEP (molecular electrostatic potential) distributions over the whole *PAS* molecular structure, that indicated its high adsorption power over *X-65* surface with construction of a protective layer against the corrosive particles^113,114^. The *PAS* COSMO field in Fig. 11. presented carbonyl group, amide group and N-atoms with red color has high electron density resulting in *PAS* reactivity enhancement with *X-65* surface which demonstrated high mitigation potency of *PAS*^115–117^. According to HOMO and LUMO energies (${E}_{HOMO}$ and ${E}_{LUMO}$), several quantum indices in Table 9 were calculated as follow:

**Table 9:** Computed Quantum chemical parameters of *PAS* inhibitor.

*Inh*	${E}_{HOMO}$ (eV)	${E}_{LUMO}$ (eV)	∆*E*_gap_ (eV)	*I* (eV)	*A* (eV)	$\sigma$ (eV)	$\eta$ (eV mol^-1^)	${E}_{\mathrm{b}\to \mathrm{d}}$ (eV mol^-1^)	$\chi$ (eV mol^-1^)	$\Delta N$
*PAS*	-0.1766	-0.0603	0.11637	0.1766	0.0603	17.186	0.0581	-0.0145	0.1185	59.13

23 $$\Delta {E}_{gap}={E}_{LUMO}-{E}_{HOMO}$$

24 $$I=-{E}_{HOMO}$$

25 $$A=-{E}_{LUMO}$$

26 $$\chi =\frac{-({E}_{HOMO}+{E}_{LUMO})}{2}$$

27 $$\eta =\frac{\Delta {E}_{gap}}{2}$$

28 $$\sigma =\frac{1}{\eta }$$

29 $${E}_{\mathrm{b}\to \mathrm{d}}=\frac{-\eta }{4}$$

30 $$\Delta N=\frac{({\chi }_{Fe}-{\chi }_{Al-Sb})}{2\left({\eta }_{Fe}+{\eta }_{Al-Sb}\right)}$$

Here, $\Delta {E}_{gap}$, *I*, *A*, and $\chi$ are energy gap, molecular ionization potential, electron affinity, and electronegativity. $\eta ,$ σ, ${E}_{\mathrm{b}\to \mathrm{d}}, \text{and } \Delta N$ denote global hardness, chemical softness, energy of back donation and fraction of electron transfer. Besides, ${\eta }_{Fe}$ and ${\chi }_{Fe}$ are 0 eV/mol and 7 eV/mol respectively^55,118^. The values of ${E}_{HOMO}$ and ${E}_{LUMO}$ in Table 9 demonstrated *PAS* adsorption onto *X-65* surface via electron donation-acceptance process^111,119,120^. Also, the diminished worth of $\Delta {E}_{gap}$ as in Table 9, confirmed the high reactivity of the investigated *PAS* and its ease interaction with *X-65* surface consequently inhibited *X-65* effectively which was also assured from *I* and *A* lower values^74,121^. It was noticed that in Table 9, *A* value < *I* value which proposed methodology for chemical bonds construction between *PAS* and vacant d-orbitals of Fe via electron-donation process^118^. In addition, $\eta$ and σ vacant verified *PAS* adsorption over *X-65* accompanied with *PAS*/*X-65* complex formation. The high positive $\Delta N$ value revealed *PAS* competence to interact with Fe surface through electro-donation process and construction of a strong barrier layer insulation *X-65* surface from the destructive particles^122,123^. All these explanations proved the mitigation competence of the studied *PAS* for *X-65* corrosion in acidic 1 M HCl environment.

#### MCs (Monte Carlo simulation)

**Fig. 12:**
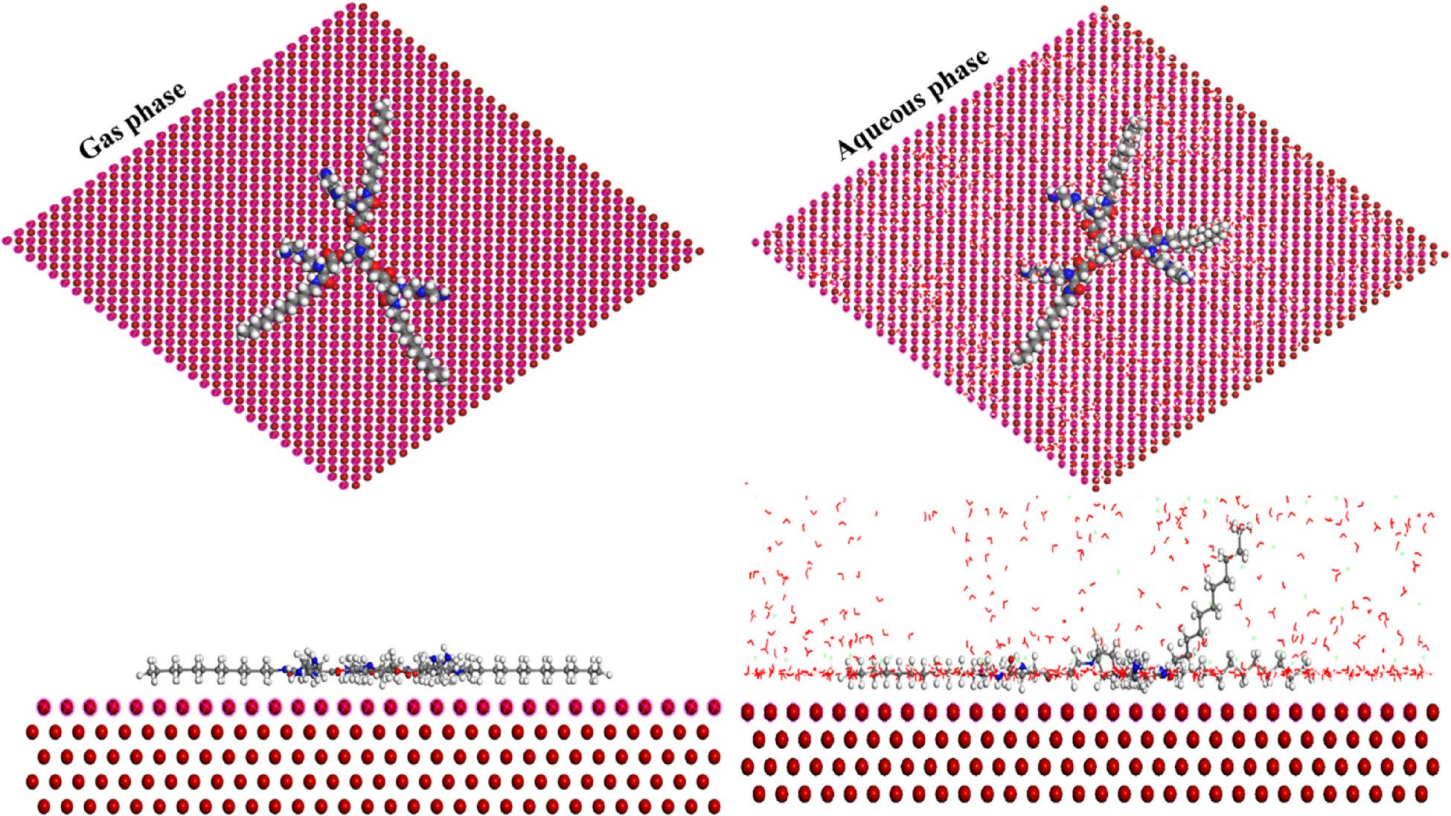
Equilibrium adsorption configuration of the studied *PAS* in both gas and liquid phases on Fe (110) obtained by MCs.

*MCs* was employed for *PAS* adsorption prediction onto Fe (110) surface as depicted in Fig. 12, showing the optimal configuration of the investigated *PAS* adsorbed over *X-65* surface in both isolated (vacuum) and liquid (H_2_O, H_3_O^+^, and Cl^-^) phases. The existence of *PAS* onto Fe (110) surface with parallel alignment with *PAS* active centers oriented towards *X-65* surface reflected the high interaction between *PAS* and Fe surface foaming an insulation film layer shielding *X-65* surface against the destructive species^55,124^. This fact proved the *PAS* adsorption power and its mitigation effect with distinguished role in *X-65* corrosion control. Also, the data obtained in Table 10 containing the adsorption energy (*E*_ads_), rigid energy (*E*_rig_), deformation energy (*E*_def_), and energy ratios for the corrosive particles raveled *PAS* inhibitive effect. As noticed, the *PAS* with negative *E*_ads_ value which indicated that, the studied *PAS* adsorbed onto *X-65* surface spontaneously with formation of an inhibitory layer^125,126^. The *E*_ads_ of the examined *PAS* in aqueous phase was very low relative to those of the corrosive species which demonstrated construction of *PAS*-Fe complexes accompanied with a defensive film formation via replacement process of the destructive particles over *X-65* surface with the adsorbed *PAS* molecules^127^. Moreover, the *E*_ads_ value in the simulated aqueous phase compared with that value in vacuum phase verified the strong adsorption capacity of *PAS* inhibitor with chemical bond formation between the oriented *PAS* active centers as N-atoms, O-atoms, carbonyl, and amide groups with the unoccupied d-orbitals of Fe^128^. The above annotations from *DFT* and *MCs* data exhibited the vital role of *PAS* inhibitor in *X-65* protection which was matched with the experimental studies.

**Table 10:** The outputs energies calculated by MCs for *PAS* in gas and simulated liquid phases on Fe (1 1 0).

Phase	*E*_T_ (kJ/mol)	*E*_ads_ (kJ/mol)	*E*_rig._ (kJ/mol)	*E*_def._ (kJ/mol)	(dE_ads_ /dNi) (kJ/mol)			
					*PAS*	H_2_O	H_3_O^+^	Cl^-^
Gas phase	-759.001	-788.951	-792.787	-3.836	-785.951	–	–	–
Liquid phase	-25,440.22	-43,150.75	-26,038.39	-17,112.35	-788.809	-29.267	-157.866	-139.785

### Surface analysis

**Fig. 13:**
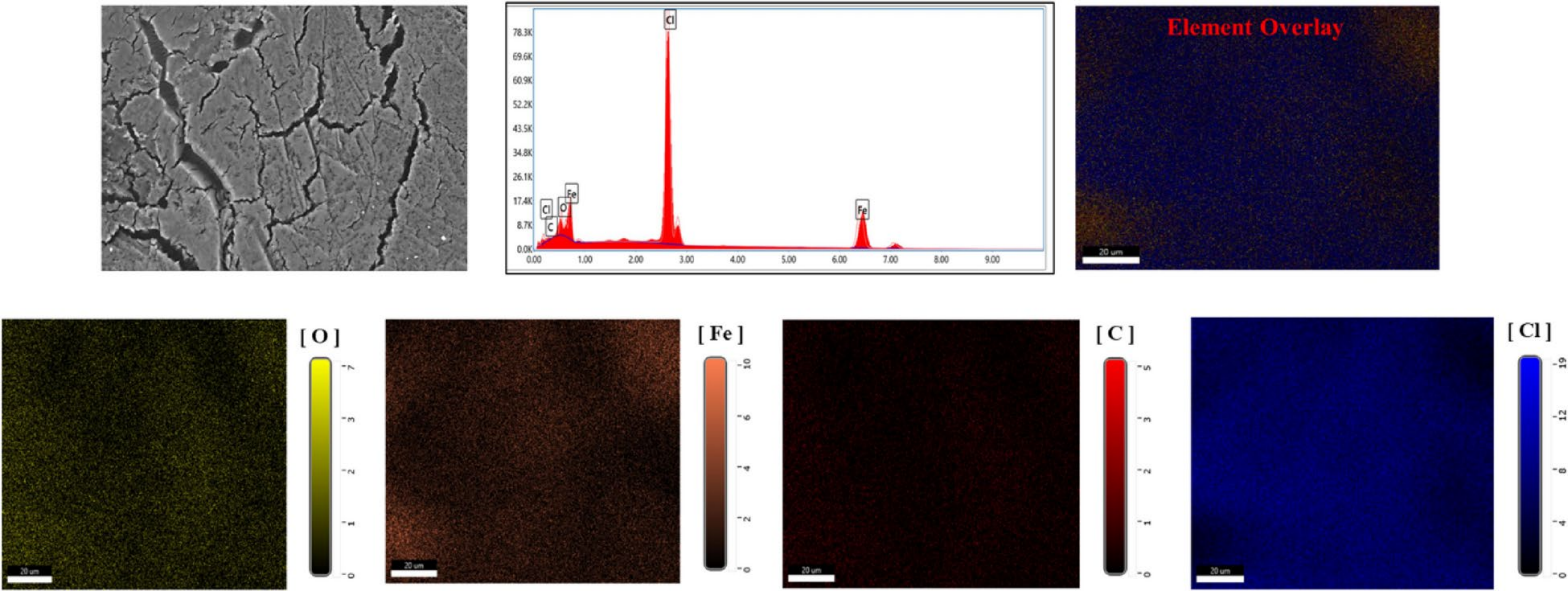
*SEM* and *EDX* mapping for *X-65* in blank solution (1 M HCl) after 6 h immersion.

**Fig. 14:**
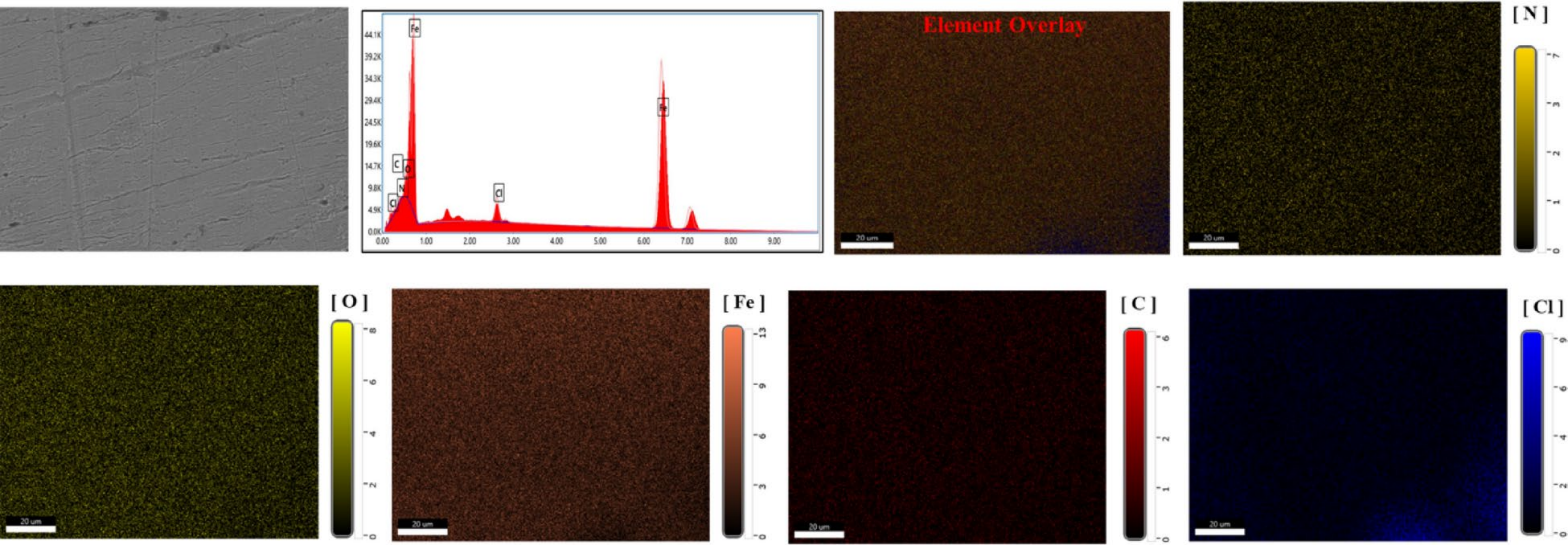
*SEM* and *EDX* mapping for *X-65* in 1 M HCl free and containing *PAS* inhibitor after 6 h immersion.

**Fig. 15:**
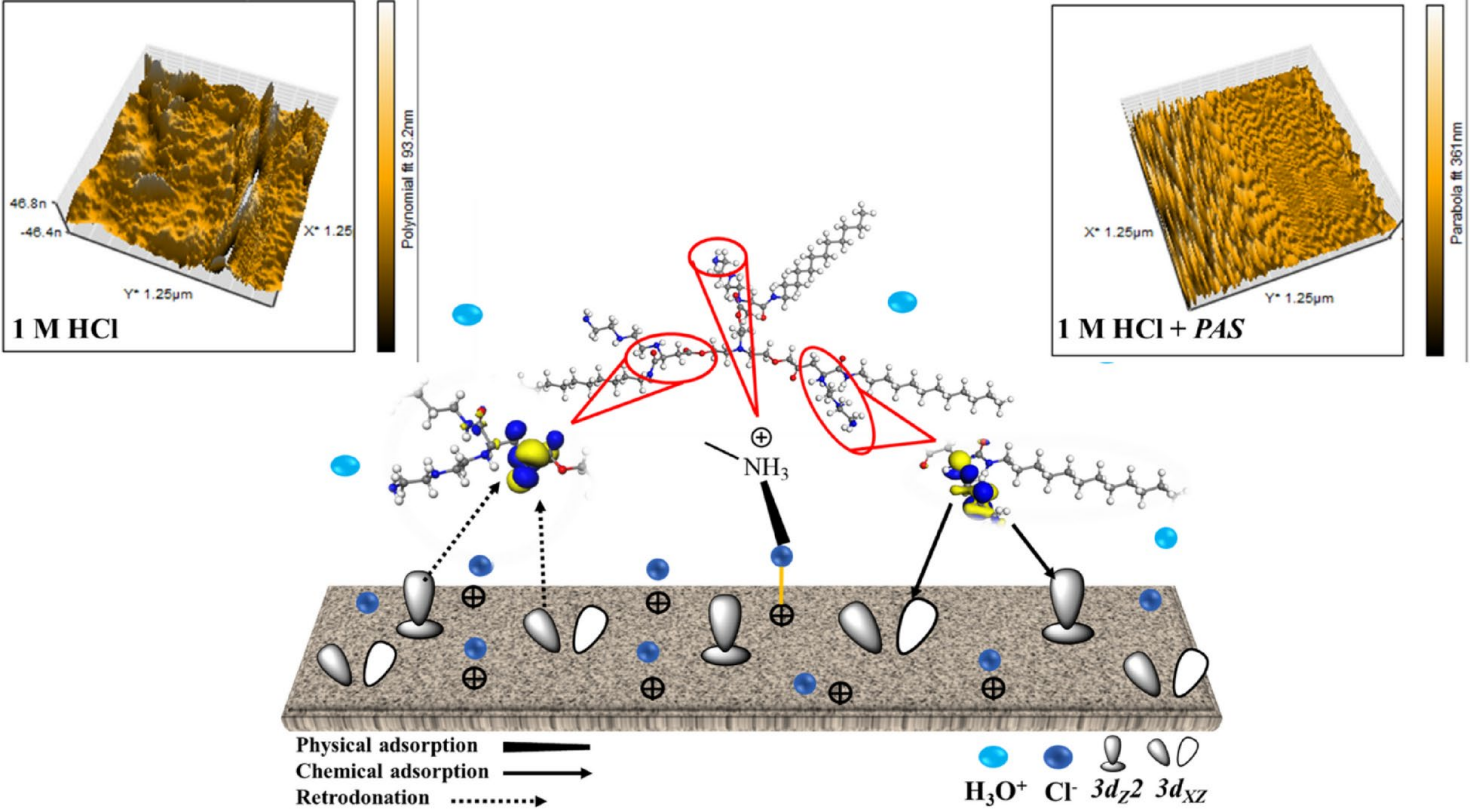
*AFM* of *X-65* in 1 M HCl free and containing *PAS* inhibitor after 6 h immersion with the simulated adsorption mechanism.

SEM analyses of *X-65* surface both before and after addition of 1000 µM of the *PAS* to the corrosive media can be seen as described in Figs. 13 and 14. The findings highlighted the *X-65* corrosion process in the untreated solution, where the surface exhibited significant damage and roughness due to HCl destructive effect^97,129^. In contrast, a markedly improved in *X-65* surface was observed following the introduction of *PAS* inhibitor which can be attributed to its mitigation power and construction of barrier defensive layer of *PAS* molecules, which suppressed the interaction between *X-65* surface and the corrosive environment^98^. *EDX*-mapping was carried out to identify the elementary analysis of the *X-65.* The observed results obtained from Figs. 13 and 14 confirming the annotations presented by *SEM* images showing *X-65* immersed in the untreated solution with destructive surface and corrosion products primarily consists of iron oxides and chlorides, with chloride and oxide weight percentages (*wt*%) 48.26% and 12.91%, respectively. In addition, the Fe-peak with weight percentages 34.76%, reflecting the existence of iron in the untreated corrosive solution with soluble ions forms which facilitated their reaction with chlorides and oxides in the corrosive surrounding and produced corrosion products of iron oxides and chlorides^21,130^. While, the notably decline in chloride content with weight percentage 3.29% after the *PAS* addition which can be explained by the adsorption process of *PAS* molecules onto *X-65* surface, that can also be verified by the appearance of nitrogen peak weigh percentages 1.12%. This confirmed the formation of the adsorption layer composed of smooth iron oxides and the *PAS* compound on the *X-65* surface^97,129^. It is worth noting that, the intensity of the Fe-peak with improved and *wt*% of Fe was 90.16% after the addition of *PAS*, indicating construction of a defensive barrier layer shielding *X-65* surface against the destructive agents^131,132^. AFM with 3D images in Fig. 15 showed different 3D images of *X-65* surface prior to and following the *PAS* inhibitor addition showing corroded *X-65* surface with average roughness 54.61 nm in the unprotected solution with higher surface roughness owing to HCl aggressive action^133^. While, after the addition of *PAS* inhibitor, *X-65* surface morphology improved due to formation of adsorbed protective layer of *PAS* molecules decreasing average surface roughness to 18.27 nm^134^**.** All the above annotations highlighted the protection capacity of the investigated *PAS* for *X-65* immersed in corrosive acidic environment (HCl) via formation of an insulation layer, covering more *X-65* surface area from the corrosive agents subsequently suppressed *X-65* corrosion rate. Additionally, the adsorption mechanism of *PAS* inhibitor with various adsorption modes was simulated and represented in Fig. 15.

### Inhibition mechanism

The prepared *PAS* inhibitor declines the destructive effect of HCl solution via its adsorption process over *X-65*, suppressing its corrosion rate via decreasing the contact between the corrosive particles and *X-65* surface by blocking its active sites. The functional groups of the star-shaped polyamine contribute to adhesion primarily through strong, multivalent chemisorption, which is directly responsible for its corrosion inhibition performance. The lone electron pairs on the N-atoms in the primary and secondary amine groups can form coordinate covalent bonds with the empty d-orbitals of iron atoms. This creates a persistent, adsorbed monolayer. Physical adsorption via electrostatic attraction between the charged sites in the *PAS* structure and the *X-65* negative sites. Also, the prepared *PAS* showed a good surface property due to its star-shaped structure, which combines hydrophobic long alkyl chains with a hydrophilic polyamine backbone. The multi-armed, 'star-shaped’ architecture is critical here. It allows the molecule to: bind at multiple sites across the steel surface, creating a more stable and densely packed barrier, cover a wider surface area per molecule, enhancing efficiency, form a more uniform and resilient hydrophobic film that blocks the diffusion of the corrosive particles to the steel surface. This strong, multi-point adhesion physically and electrochemically passivates the surface. It raises the energy barrier for the anodic (iron dissolution) and/or cathodic (H_2_ evolution) reactions. The obtained data reflected the mixed nature of inhibition (physical and chemical). This feature enhanced the corrosion mitigation capabilities of *PAS* for *X-65*. Finally, MCs of *PAS* designated the horizontal orientation of the studied *PAS* inhibitor over the Fe (110) surface, which increased surface coverage percentage of *X-65*, consequently declined the rate of the corrosion process. Also, the comparison between the inhibition efficiency of the prepared *PAS* at optimum concentration (1 × 10^–3^ M) with other similar inhibitors with higher concentration (= or > 1 × 10^–3^) M as in Table 11 reflected *PAS* superior mitigation potency.

**Table 11:** Comparison between the inhibition efficiency of *PAS* and other investigated inhibitors for *CS* in 1 M HCl solution.

Compound	Conc. M	EIS *η %*	PDP* η %*	R
I	1 × 10^–3^	68.8	68.4	^135^
II		82.6	81.6	
III		86.6	85.5	
S2	7 × 10^–4^	84.48	91.83	^136^
S3		81.48	90.07	
SH600	1 × 10^–4^	63.55	63.18	^137^
SH1000		67.24	70.29	
SH1500		75.7	72.89	
NIS I	5 × 10^–2^	82.69	68.91	^104^
NIS I		80.81	65.55	
CDEA	2 × 10^–2^	77.9	73.7	^138^
*PAS*	1 × 10^–3^	95.46	94.63	Present study

## Conclusion

The present research investigated the inhibition performance new star like shape *PAS* surfactant for *X-65* steel immersed in 1 M HCl solution. The findings bolstered that, *PDP*, *EIS*, *SEM*, *EDX*-mapping, AFM analyses established that *PAS* effectively retarded the corrosion process of *X-65* steel with an inhibition efficiency about 96% at a concentration of 1000 µM. *PDP* data reflected the mitigation power of *PAS* with mixed-type of inhibition through decline both metal dissolution and H_2_ evolution reactions. Also, *EIS* results exhibited a notable enhancement in *R*_*P*_ values after the introduction of the studied *PAS* inhibitor which can be attributed to its adsorption process upon *X-65* surface which obeyed Langmuir adsorption isotherm. Finally, *DFT* highlighted the electronic properties of *PAS* and its adsorption over *X-65* surface via electron donation-acceptance process. Besides, *MCs* suggested that the examined *PAS* adsorbed nearly parallel to Fe (110) surface.

## Data Availability

All data generated or analyzed during this study are included in this manuscript.
